# Scoping Review on Ageism against Younger Populations

**DOI:** 10.3390/ijerph18083988

**Published:** 2021-04-10

**Authors:** Vânia de la Fuente-Núñez, Ella Cohn-Schwartz, Senjooti Roy, Liat Ayalon

**Affiliations:** 1 Demographic Change and Healthy Ageing Unit, Department of Social Determinants of Health, World Health Organization, 1202 Geneva, Switzerland; 2 Department of Public Health, Ben-Gurion University of the Negev, Beer-Sheva 8410501, Israel; ellasch@bgu.ac.il; 3 Louis and Gabi Weisfeld School of Social Work, Bar Ilan University, Ramat Gan 5290002, Israel; roys3@miamioh.edu (S.R.); liat.ayalon@biu.ac.il (L.A.)

**Keywords:** ageism, youthism, discrimination, stereotype, prejudice, scoping review

## Abstract

Systematic efforts have been carried out to study ageism against older populations. Less is known about ageism against younger populations, including how it is defined, how it manifests, its effects, and how it can be addressed. A scoping review was conducted aimed at identifying available evidence on these topics. A comprehensive search strategy was used across thirteen databases, including PubMed, EMBASE, and CINAHL. Records were screened by two independent reviewers. Data extraction was done by one rater and independently reviewed by a second rater. Of the 9270 records identified, 263 were eligible for inclusion. Most of the evidence focused on the manifestation of ageism (86%), followed by a focus on the determinants of ageism (17%), available interventions to address ageism (9%), and the effects of ageism (5%). This study points to the inconsistent terminology used to describe ageism against younger populations and the relatively limited theoretical rationale that guides existing studies. It also highlights key research gaps and points to the strengths of existing research.

## 1. Introduction

Ageism, defined as the stereotypes, prejudice, and discrimination towards people on the basis of their age [1] can affect any age group. It can be directed towards others or towards oneself (e.g., self-directed ageism). To date, most of the existing literature on the topic has focused on ageism as it affects older adults, including several systematic reviews on the determinants, impact, and measurement of ageism and on available strategies to reduce this phenomenon [2,3,4,5]. These same issues, however, have not been adequately or systematically explored in relation to younger age groups, including children, young adults, and middle-aged people [6]. This is particularly concerning given available data from several large scale social surveys showing that younger people, more so than other age groups, report exposure to discrimination based on age [7,8], and elicit more negative feelings than older adults among the public [9]. Moreover, past research has stressed how common it is for younger people to have to take on precarious, unstable jobs [10] or unpaid internships [11], and how they tend to be most affected during financial crises [12]. As ageism is directed towards any age group and has shown to have a negative impact on older adults [5], it is essential to review and summarize existing research concerning ageism directed towards younger populations.

To explore the current knowledgebase concerning ageism as it affects younger people, this study conducted a scoping review of available literature regarding ageism against younger populations, defined as those under 50 years of age for the purpose of this study. The rationale for including this age cut-off is that most evidence synthesis exploring ageism has included studies where people over the age of 50 were the target population [2,3,4,5]. As past research has mainly addressed ageism in the second half of life, the scope of this review was on ageism towards this broader category of people under the age of 50, rather than towards smaller age groups. Where specific aspects applied only to a given age group or life stage (e.g., adolescence), this has been highlighted. A scoping review methodology was used as this type of knowledge synthesis is particularly useful when: (a) no prior synthesis has been undertaken on the topic; (b) studies have employed a range of data collection and analysis techniques; and (c) a narrow review question cannot be defined [13,14,15]. Scoping reviews are intended to provide analytical interpretation of the literature, identify key concepts, and types of evidence and may also provide the background for full systematic reviews or identify areas where existing research is limited or lacking [16,17,18,19].

This review was guided by the following five questions: (1) “What terms are used to refer to ageism towards younger populations?”, (2) “How prevalent is ageism towards younger populations and how does it manifest?”, (3) “What are the determinants of ageism towards younger populations?”, (4) “What are the effects of ageism towards younger populations?”, and (5) “What strategies exist to tackle ageism towards younger populations?”. The present study aims to serve as a platform for future research and policy on the topic of ageism against younger populations by summarizing existing evidence and pointing to potential knowledge gaps.

## 2. Materials and Methods

The scoping review methodology was based on the framework outlined by Arksey and O’Malley and ensuing recommendations [14,20,21]. The review included the following key phases: (1) identifying the research question(s), (2) identifying relevant studies, (3) study selection, (4) charting the data, and (5) collating, summarizing, and reporting the results. The conceptual framework of ageism used in this scoping review was the one proposed by Iversen and colleagues [22].

### 2.1. Identifying Relevant Studies

The search strategy was developed by VFN in consultation with an information specialist. The primary search terms focused on the concepts of ageism (e.g., ageism, ageist) and younger populations (e.g., young population and middle-aged). The Boolean term “AND” was used between these two primary concepts. Additional terms used in the literature to specifically refer to ageism towards younger populations were also included (e.g., “youthism”, “kiddism”, and “adultism”). See Table S1 for the search strategy that was used for PubMed and translated to other databases.

The initial search was conducted on 29 May 2019, in 13 electronic databases, including Campbell, CINAHL, Cochrane, DARE, EBSCO, EMBASE, GMI, GreyLit, OpenGrey/GreyNet, ProQuest, Prospero, PsycInfo, and PubMed. No year restrictions were applied. The databases were selected to be comprehensive and to cover a broad range of disciplines.

A snowball search was conducted to identify additional records by using Google Scholar’s “cited by” and “related to” functions for each of the articles included in the original search [23]. The results of all searches were entered into the Covidence software program for reviews [24] and duplicates were removed.

### 2.2. Study Selection

**Figure 1:**
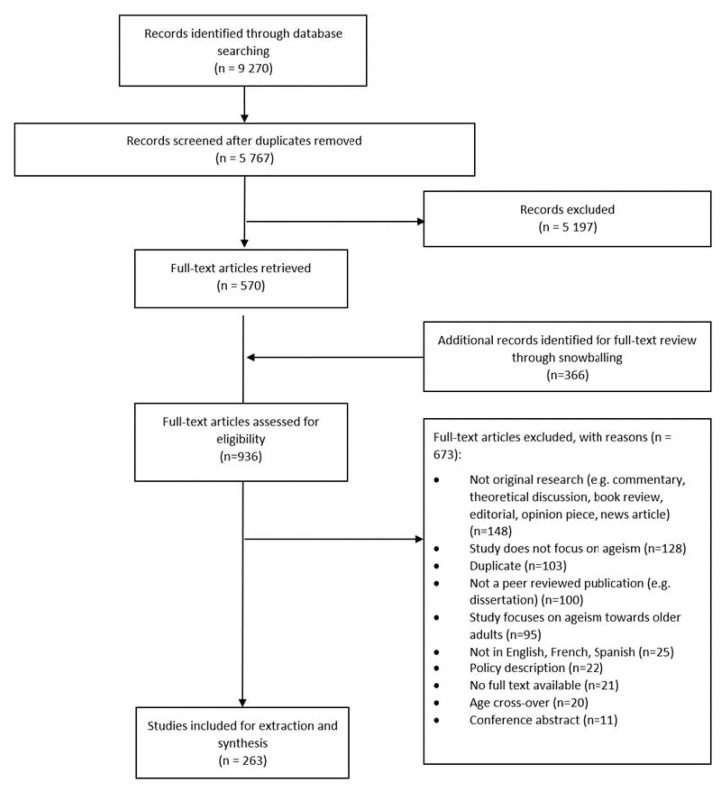
Study selection flowchart.

A two-stage screening process was used to assess the relevance of studies identified in the search. First, titles and abstracts were screened by two independent reviewers (ECS and SR) with disagreements resolved through consensus with a third reviewer (VFN or LA). The full text of shortlisted articles was subsequently reviewed by two independent raters among the authors with disagreements resolved by a third reviewer. Eligible studies met the following inclusion criteria: (a) full text available in English, Spanish, or French with non-English language articles having English abstracts available, (b) peer-reviewed publication including both quantitative and qualitative research, and (c) research that focuses on ageism towards younger people (defined as those aged 50 or below). For articles that could not be obtained through institutional holdings available to the authors, attempts were made to contact the source author or journal for assistance in procuring the article. Opinion pieces, book chapters, theoretical discussions, and articles providing a description of a policy or law with no empirical findings were excluded. Studies including a study sample with an age cross-over (e.g., participants between ages 45 and 55) were also excluded. The search flow is represented in Figure 1.

### 2.3. Charting the Data

A draft charting table based on the Cochrane data extraction tool was used. It included the following overarching themes: key study identifiers, study methodology, study sample characteristics, and study details/results. The charting table was piloted and refined by VFN and LA who independently charted the first ten studies to determine whether the approach to data extraction was consistent with the research questions and purpose [20,21]. The remaining studies were charted by one author (among all authors), with another author independently reviewing and confirming the data extraction. Disagreements were discussed among the authors until a consensus was reached.

### 2.4. Data Summary and Synthesis

The data were compiled in a single spreadsheet using Microsoft Excel for validation and coding. Data that met all inclusion criteria were summarized descriptively and a narrative synthesis was conducted to respond to each of the predefined research questions [25].

## 3. Results

**Figure 2:**
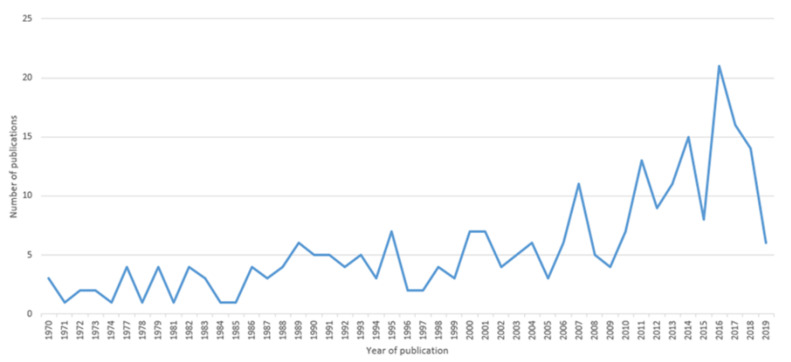
Publications per year on ageism against younger populations.

A total of 263 articles were included, published between 1970 and 2019 (see Figure 1). As can be seen in Figure 2, the number of articles increased substantially from an average <5 for the first two decades of research on ageism to over 20 articles in 2017.

### 3.1. Study Characteristics

A total of 60 different countries are represented in the studies included in the scoping review. The vast majority of studies were conducted in the United States of America (*n* = 145) followed by the United Kingdom (*n* = 25), China (*n* = 17), Germany (*n* = 15), Australia (*n* = 12), and Canada (*n* = 11). Only 22 studies were conducted in two or more countries, simultaneously.

University and college students were the most common study samples (*n* = 106). Other study samples included, employers and HR professionals, employees in various sectors, community dwellers, preschool children, primary and secondary school students, older adults living in nursing homes or assisted living communities, and people participating in intergenerational programs. In the vast majority of studies, women were either equally represented or more represented than men. The target age groups studied ranged between 0 and 49 years, with most studies looking at various age groups. Only a handful of studies looked specifically at populations below 18 as the target of ageism (*n* = 26). Over half of the studies examined younger populations against older populations (50 years old and above) to demonstrate ageism (*n* = 175).

The research evidence in this area mainly comes from quantitative studies (*n* = 217). A relatively smaller number of studies used qualitative methodology (*n* = 53), with some studies (*n* = 7) using both quantitative and qualitative methods.

**Figure 3:**
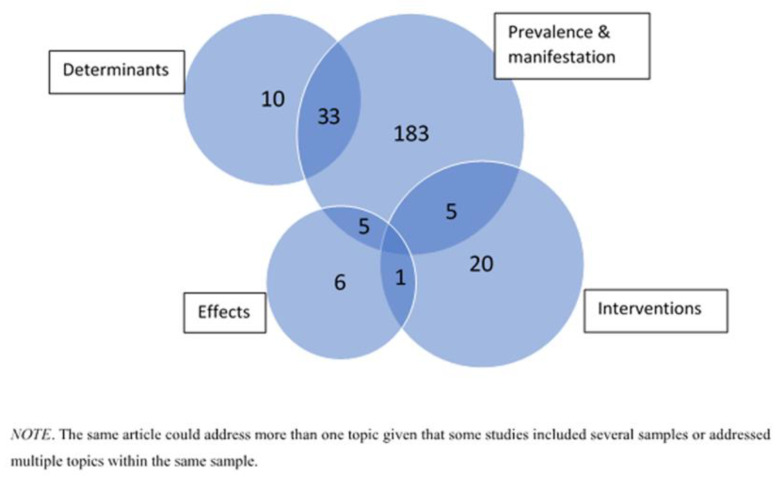
Topics addressed by the studies included in the scoping review.

Of the 263 publications included in the scoping review, most focused on the manifestation of ageism (86%), followed by a focus on the determinants of ageism (17%), interventions to tackle ageism (9%), and effects of ageism (5%) (see Table 1). Many of these publications explored multiple questions as illustrated in Figure 3. Table S2 presents the main characteristics of the 263 studies, organized alphabetically.

### 3.2. First Research Question: What Terms Are Used to Refer to Ageism towards Younger Populations?

The articles used a variety of terms to refer to ageism towards younger populations, including not only ageism [71,140,206], but also reverse ageism [219], age-based bias [37,40,68,69,78,158,196], childism [194], and adultism [96,101,111,211].

Although initially defined as only regarding biases against older individuals, the term ageism is increasingly being used to apply to individuals across the spectrum of age, both old and young [178,252]. In turn, reverse ageism is generally used to refer to ageism directed at younger adults, who tend to be broadly defined as people in their 20s and 30s [219], and childism is used in the literature to specifically refer to a kind of unique bias against children [194].

The term adultism is used to refer to the stereotypes, feelings and behaviors of adults towards children and youth, which are based on the assumption that youth and children are naïve, inexperienced, or incompetent and that adults know better and are thus entitled to act upon them without their agreement [96,211]. Adultism represents the structural power that adults have over children in our society. Adultism can manifest in many ways including over-victimizing youth, infantilizing youth, romanticizing youth, and tokenizing youth and is often reinforced by social institutions, laws, and customs [101,111].

### 3.3. Second Research Question: How Prevalent Is Ageism towards Younger Populations and How Does It Manifest?

#### 3.3.1. The Overall Prevalence and Manifestation of Ageism towards Younger Populations

Only two of the studies included in the scoping review aimed at estimating the overall prevalence of ageism towards younger populations (e.g., did not focus on its prevalence in a specific sector like employment). Using the European Social Survey data, these two studies estimated the prevalence of age-based discrimination in 29 countries, reporting highest levels of perceived age discrimination among younger respondents, and a U-shaped distribution of age-discrimination, with greater levels among older and younger adults than middle-aged people [6,9].

Other studies looking at ageism outside of any one setting or institution (*n* = 93) explored the ways in which it manifests, including whether younger populations trigger specific stereotypes and prejudice in relation to their personality, emotions, performance, mental and physical capacity, vitality, physical appearance, and sexuality. For example, one study examined the content and consistency of age stereotypes across more than 20 countries, finding important differences in the ratings of adolescents, adults and older adults with regards to traits such as impulsivity, activity, and openness. The study found that raters across countries tended to share similar beliefs about different age groups with adolescents seen as impulsive, rebellious, and undisciplined [97]. A meta-analysis of papers on attitudes towards younger and older people, found that younger people were rated less stereotypically, seen as more attractive and more competent, and were evaluated more favorably than older adults [46].

#### 3.3.2. Ageism towards Younger Populations in Communication and Social Relations

A total of 21 different studies examined the manifestation of ageism towards younger people in communication and social relations, including intergenerational relations. Research shows that younger adults tend to interact with same age peers [103,155,168,186,199,244] and to hold positive interpersonal attitudes towards their own age group [134,155,205,244].

Research also found that younger people felt being patronized in interpersonal relations with older adults [137] and that both age groups rely on age stereotypes in interpersonal relations [171]. When opportunities for intergenerational relationships and shared spaces arose, age-based stereotypes were less likely to be applied [112].

Results concerning older adults’ attitudes towards younger people were mixed with some studies suggesting that older adults prefer younger people [98,103] or hold positive attitudes towards younger populations [200]. Other studies did not find this age-based preference or even reported negative stereotypes and prejudice from older persons towards younger persons [106].

#### 3.3.3. Ageism towards Younger Populations in Specific Institutional Settings or Sectors

The vast majority of the literature exploring the manifestation of ageism has focused on its occurrence in specific settings or sectors, particularly in relation to employment (*n* = 75), health and social care (*n* = 26), power and politics (*n* = 16), and justice (*n* = 9). As illustrated in Table 2, a few other sectors have also been studied but have received relatively little attention. The rest of this section will focus on the four sectors that have received most attention.

Employment: The articles that have explored ageism in employment have mainly looked at its manifestation in recruitment processes or in the workplace, once the person is employed. The general conclusion that can be drawn from the studies looking at ageism in recruitment is that younger populations have increased access to interviews and are favored in hiring decisions relative to both middle-aged adults and older adults [51,61,71,72,88,109,114,123,124,139,146,209,210]. However, variability in response exists and has been attributed to a variety of factors related to the characteristics of the workplace and the candidate [32,128,195,196,221]. Whilst these studies suggest that younger adults are less likely to experience discrimination in hiring processes, one study that looked at the intersection between age and sex did find significant discrimination against younger women applying for high skilled administrative jobs [212].

Age bias seems to manifest more crudely once younger adults are in the workplace. Younger workers report feeling more discriminated and disadvantaged because of their age than middle-aged and older people [183,238] and one study conducted in the UK showed that 1 in 3 younger workers reported experiences of age discrimination [178]. Other studies report that even if all age groups are affected by age discrimination in the workplace to some degree, younger adults, especially younger women, are most affected, especially in terms of pay and benefits [115,131,179]. In Iceland, this discrimination may even amount to child labor laws violations [116].

Younger workers report not feeling valued, receiving belittling comments and being generally perceived as incompetent because they look young, and receiving fewer development opportunities [183,193,219]. Another article makes a distinction between enacted stigma whereby a person makes explicit comments about a participant’s age, and felt stigma where the participant is made to feel uncomfortable and self-conscious about age, noting that whilst both older and younger workers report instances of felt stigma, only younger workers report instances of enacted stigma [189].

Available studies looking at ageism in performance evaluations generally conclude that there is no discernable age bias in ranking similarly performing employees by employers [113,228] or clients [53,65]. However, this may be dependent on the specific employment sector [226]. A literature review also found that age might be less important than individual skill and health on evaluation of job performance [216]. Whether there is an age effect on corrective actions taken to improve performance is also unclear: two studies showed that younger workers may be more likely than older adults to get recommended for training to improve performance [33,105] with another one finding no effect of age [85].

Age discrimination does seem to affect employees’ dismissal. One study conducted in Australia found that the dismissal of younger employees (15–24 years old) was most associated with bullying, harassment, and taking personal leave. Young men, compared to young women, were disproportionately likely to report allegations of misconduct as preceding dismissal, while women experienced higher rates of sexual harassment and discrimination [167]. Indeed, across all ages female employees appear to be more likely to experience ageist attitudes concerning appearance or sexuality [115,166].

Other studies examined whether specific stereotypes and prejudice were directed towards younger workers relative to middle-aged and older workers. Overall, research shows that stereotypes and prejudice are different depending on the age group. For example, younger workers tend to generate less empathy and are often perceived to be less conscientious, emotionally stable, and agreeable and at the same time more creative and extraverted than older adults [50,66,119,242]. They are also rated less favorably on experience, work ethics, and stability and higher on potential for development, interpersonal skills, flexibility, and performance capacity, among other attributes [58,125,133,188]. Middle-aged workers tend to hold more negative stereotypes of younger workers [252], and younger adults report perceiving more negative age based stereotypes than older adults [122].

Health and social care: The studies that have explored manifestations of ageism in relation to health and social care have generally looked at the attitudes and discrimination of health and social care workers towards younger clients presenting with different conditions. For example, one study conducted in the US looked at the attitudes of nurses towards patients of different ages, finding that young and middle aged adults are viewed most favorably and that only children and adolescents are viewed as negatively as older people [138].

In terms of discrimination, available literature has explored age biases in access to treatment and care, and in diagnosis for different conditions, with most studies drawing a comparison between older and younger patients. Some of these studies show that younger people tend to be given preference over older adults. For example, in access to HIV antiretroviral treatment and heart transplant [127], and in terms of waiting lists [126]. There is also a significant preference for treating younger versus older patients in the vegetative state [43], and a perception that younger people deserve more psychosocial support [161]. The perceptions of social workers towards homeless people are also less harsh if the target is younger [162]. However, other research has shown that there may be considerable age biases in health and social care, which could ultimately affect health outcomes for younger people [78,132,147,176]. For example, the diagnosis and treatment options offered by doctors to patients presenting with sexual dysfunction varies depending on the age of the patient [132].

Other studies looked at the effect of intersectionality in health and social care, examining, for example the compounded effect of the age and gender of the target, or age and health status. For example, a study conducted in 2015, found that social workers were more likely to value younger heterosexual targets compared with same age gay or older clients, and recommend more aggressive treatment of terminal illnesses for these patients [164]. The gender and age of a child have also been found to affect whether a child is reported to be healthy or unhealthy in some communities and the type of treatment received, with parents reporting that females and younger children were sicker than males and older children, and females receiving more home care and fewer treatment involving cash payments [174].

The compounded effect of health and age on stigmatization and discrimination was also studied. One study found that younger adults with a mental illness may not be as stigmatized and discriminated against as other age groups [247], whereas another study showed that obese children are the population most at risk for being confronted with stigmatization [235], and yet another showed that younger people with one of several health conditions (e.g., blindness, depression, leg amputation, AIDS, and lung cancer) were more stigmatized and discriminated against than older people presenting with the same conditions [191].

Power and politics: A few of the studies included in this review looked at the status and power accorded to people on the basis of their age, showing that middle-aged adults, especially men, hold the greatest status, wealth, and power, and that younger adults are perceived to have the lowest status, wealth, and power [82,95,142].

Different qualitative studies also examined the manifestation of ageism in youth political and advocacy movements, showing that there is a tendency to doubt, deny, or dismiss the voices of youth and children [102,211], regulate their identities [111], and generally limit their efforts [101,111,232].

Other studies looked at the effect of intersectionality in power and politics, examining, for example the compounded effect of age and gender or ethnicity [160,206,225]. For example, one study looking at the experiences of a group of women labor activists participating in youth programs found that the age of the women intersected with their gender and racial identity to create systemic disadvantage and unfavorable experiences [160]. A couple of studies also looked at the existence of age bias in voting behaviors finding that ageism is a stronger factor in voting than sexism or racism, with middle-aged candidates being most preferred, followed by younger candidates [215,234].

Justice: In looking at the manifestation of ageism in justice, available studies explored whether the age of an offender or criminal or the age of the victim could affect the evaluation of a given crime and its punitiveness. The four studies that explored whether the age of an offender or criminal made a difference on the judgments made found that crimes committed by younger offenders elicited greater anger and were perceived to be more serious transgressions, and rated to deserve more severe punishment [34,89,99]. One study suggested that this age effect might not apply equally across ethnic groups [159].

The studies that examined whether the age of the victim of a crime or accident made a difference on judgments found inconsistent results. Two studies showed that transgressions were evaluated more seriously and received more severe punishment recommendations if the victim was an older adult [89,99], whereas two other studies showed no effect of age on judgments [157,231]. Another study looking at child sexual abuse of 15 versus 12 year old girls found that while attributions toward the perpetrator did not differ based on the victim’s age for women, men tended to blame the perpetrator more when victims were younger and the family more when the victim was more physically mature [56]. One study suggested that the age of the victim might be particularly influential in these decisions when the victim is perceived to be innocent [92].

The other study classified as exploring the manifestation of ageism in justice, looked at age-discrimination in employment cases in the United States, reporting that employers were most likely to win a case when the employee was younger, particularly between 40 and 49 years old [192].

### 3.4. Third Research Question: What Are the Determinants of Ageism towards Younger Populations?

A relatively large number of articles included in the review (*n* = 45) explored this research question, mainly focusing on the determinants of interpersonal ageism. A few articles also explored possible determinants of self-directed ageism.

#### 3.4.1. Inter-Personal Ageism

Characteristics of the respondent and their context: Rater age was one of the most studied drivers of ageism towards younger populations. Some studies looked at the influence or rater’s age on the preference for or stereotyping of targets of various ages [48,64,66,67,69], showing inconsistent results. Other studies explored the influence of this determinant in performance evaluations of targets of different ages by either employers [30] or clients [53,65] or in treatment decisions for younger and older patients [43]. These studies generally found no or minimal effect of rater age. Several studies also looked specifically at the effect of the rater and ratee having the same age and whether this “same age effect” was a determinant for increased prejudice and discrimination, but the results were inconsistent. For example, McNamara and colleagues found that workers rated those at the same age most highly, followed by relatively older workers, then relatively younger workers [68]. Another study found that when rater and ratee have the same age, the respondent experiences greater anger, less sympathy, and recommends more severe punishment for a thief [34].

The rater’s sex or gender was also studied as a potential driver of ageism towards younger populations with studies showing inconsistent findings. For example, two studies found no significant effect of rater’s age in ageist behaviors or stereotyping [42,51]. Two additional studies examining this driver did find that the sex of the respondent had an influence on whether the respondent had an age-biased behavior either in relation to the provision of psychotherapy to patients of different ages [55] and with regards to attributions of blame for a crime [56]. Another study also found that men were more likely than women to use age as a basis for similarity categorization, and men’s preferences clearly revealed a bias in the direction of youth [48].

Different personality traits including agreeableness, positivity, and conscientiousness of respondents were also evaluated as possible determinants of ageism towards younger people, indicating that more agreeable and positive participants were more likely to have positive evaluations for people of other age groups [36], and that high conscientiousness raters would be more likely to evaluate the performance of younger coworkers lower than that of older coworkers [47]. The religiosity of respondents [9] was also examined as a possible driver of ageism towards younger people and, in the context of therapy, respondents’ level of clinical training was assessed as a possible driver of age-biased evaluations of couples [44].

The framing of targets (i.e., the way in which they are presented) was also explored as a potential determinant of ageism. For example, one study looked at whether the framing of a specific job role influenced whether there would be an age-bias towards potential candidates [32], and another one explored whether being in a specific context and engaging in a certain behavioral activity could determine the activation of age stereotypes [29]. Other studies looked at the influence of the amount of information provided about the target, finding that the more information provided, the less likely that age bias would present [46,61]. Whether the framing involves a comparison with another age group [28] or an expectation of competition or cooperation can also result in age bias, for example, in hiring decisions [50].

Access to age-related information was also examined as a possible determinant of ageism with a study finding that under a neutral information condition, managers preferred hiring the young applicant for the low-status job, and students favored the old candidate for the high-status position. Under the age-related information condition, managers shifted to favoring the old candidate for the low-status job, and students preferred the young applicant for both the low- and high-status positions [59]. Exposure to age-biased information or stereotypes [37,62] or to disliked vs. admired young individuals [31] can also influence whether someone is ageist towards younger populations. Other studies also explored whether situations of pressure [33] or increased accountability [38,141] of the respondent in making decisions influenced the likelihood of someone being ageist towards younger people.

Contact or general exposure with younger people was studied as another possible driver of ageism with most studies finding that individuals who have increased contact with younger people, especially high quality contact, are less likely to be ageist or biased towards them [26,57]. Other literature looked at the effect of own-age exposure on age-related attitudes but found that this factor only made unique contributions to explaining better recognition memory for own-age than other-age faces [40].

The centrality of age in job prototypes was also examined as a possible determinant of age biases in selection processes for employment [54]. Following this same logic, [58] looked into the profession of the target as a possible driver or determinant recognizing that certain professions (e.g., accounting) were seen as stereotypically younger-person jobs whereas others (e.g., medicine) were considered as stereotypically older-person occupations. The influence of national and organizational culture on age stereotypes was also explored [57].

Characteristics of the target: The target’s sex was also studied as a potential driver and was generally found to affect age-biases in relation to the emotions that respondents’ perceived a girl/woman/boy/man would express [45]. It also affected performance evaluation, with young men’s high quality performance being evaluated more positively than identical performance by a young female or old man [148]. Other individual level factors affecting the target explored in the literature include the target’s health status [35] and physical appearance [41].

#### 3.4.2. Self-Directed Ageism

A few studies explored possible determinants of subjective age including health status [27], future self-views [49], and respondent’s age and sex [60]. Another study looked at whether the evaluations of own age group would influence evaluations of self in young adults, finding no significant effect, which the authors argued could suggest that age is not a salient factor when young adults evaluate themselves in comparison to others [52].

### 3.5. Fourth Research Question: What Are the Effects of Ageism towards Younger Populations?

Only 12 studies examined the effects of ageism on health, cognition, performance, overall wellbeing, and social distance. For example one study found that age discrimination alone was not associated with mental disorders in younger people but that the simultaneous reporting of age discrimination with skin color, race, and class was associated with a higher occurrence of common mental disorders [86]. Another study found that exposure to stereotypical information regarding memory capacity and age had a negative impact on memory performance across individuals with lower education [258]. When younger people see themselves under the control of powerful others, exposure to negative age-relevant stereotypes can have a positive impact on their performance, which suggests that younger adults’ reactivity to age-relevant threats is in the opposite direction of the damaging effects observed in older adults [260]. This is based on the hypothesis that younger adults would show efforts to disconfirm that they are “(too) young and inexperienced” by performing well on tasks described as requiring life experience. The impact of perceived age discrimination on wellbeing is contested with one study finding that discrimination does not harm wellbeing [259] and another finding that it does have harmful effects on the subjective wellbeing of all persons regardless of their age, but especially middle-aged people [261]. The possible effect of dual age identification (based on age group and generation) on psychological wellbeing was also studied but the findings suggested that it only has an effect on older adults (i.e., older adults’ identification with their age group led to lower levels of psychological well-being and the reverse was true when they identified with their generation), not having any significant effect on younger or middle-aged adults [263].

Perceived ageism can also have an effect on satisfaction with coworkers [224], on employees’ affective commitment to their organization [238,262], and on work identities, including how perceived discrimination could result in younger workers consciously portraying themselves as older and less feminine through dress, speech, and behavior [264].

Perceptions of age-biased communication behaviors (i.e., accommodative or non-accommodative behavior, avoidant behavior, and respectful behavior) were also found to have an impact on younger adults’ self-esteem and life satisfaction [200].

### 3.6. Fifth Research Question: What Strategies Exist to Tackle Ageism towards Younger Populations?

A total of 24 articles examined strategies to tackle ageism towards younger populations. Most of the strategies examined in the literature focus on intergenerational activities (*n* = 16) and generally find that these result in a reduction of negative attitudes towards younger populations, improved feelings of communion between generations, increased respect and understanding and sense of comfort with intergenerational interaction [103,112,265,267,269,270,271,272,274,275,277,278,279]. Still, two studies that used quantitative [268] and qualitative methodologies [106] found a very small effect or no effect of intergenerational programs in reducing ageism and a third study was unable to derive meaningful conclusions [266]. The types of activities that younger and older generations engaged in part of these programs was very diverse ranging from playing videogames [269], instrument playing interventions [267], and sharing life stories [106] to the joint preparation of a photographic poster exhibition [103] or glove puppets [275], among others.

Policies and laws have also been explored empirically as possible strategies to tackle ageism towards younger people, though to a very limited extent. For example, one study looked at the effects of legislation prohibiting age discrimination on the age characteristics specified in job advertisements, recruiters’, and employers’ references to age in the recruitment process, and people’s perceived age references in past job interviews. The study found that only 5.9% of all job ads appeared to be open to all age groups, recruiters asked about age of pseudo-applicants in 18% of cases, employers still acknowledged asking about age in approximately 32% of cases and 44% of respondents remembered being asked about their age in interviews [88]. Still, the interpretation of these findings is challenging because there was no baseline data. Another cross-sectional study explored whether equity norms reduced age discrimination, finding that such norms can help increase enthusiasm for both young and old applicants but did not necessarily reduce age-based hiring discrimination [281]. One study also found that proportional representation electoral systems favor the election of younger members of parliament even after controlling for multiple alternative explanations [282].

Other strategies have been studied, which can prevent age stereotypes’ influence on behavior. For example, one study found that self-awareness manipulations could help prevent age stereotypes from entering into deliberation and influencing social judgments [280]. Another study found that direct debiasing in the form of explicit informative warnings can reduce the influence of age stereotypes on performance appraisal and that indirect debiasing can influence hiring decisions, though this was mainly studied in relation to older candidates [109].

A final set of strategies that has been studied includes interventions that can affect how an individual copes with experiences of ageism. For example, Finkelstein and Zacher examined whether having a higher self-concept could influence how a person reacts to specific stereotypes [122]. In turn, Worth described different strategies used by young women to cope with the intersections of ageism/sexism in the workplace, explaining that while some employ conscious strategies to be “taken seriously” through dress, small talk, and even taking on stereotypical traits of masculinity to be recognized as competent, others explicitly confront inequality through “girlie feminism” with a profemininity work identity that challenges the masculine-coded norms of how a successful workplace operates and what it looks like [264].

Two broader strategies referred to in the literature to reduce ageism relate to the use of participatory action research in schools, given the role that sociocultural context plays in creating spaces where students gain skills to become change agents within their context [273]. Similarly, another study examined how partnering with youth in planning community based activities can enhance younger adults’ confidence in voicing their concerns and contributing [276].

## 4. Discussion

This article aimed to identify and summarize available literature on ageism towards younger populations. Research on ageism has increased since the word ageism was first coined by Butler in 1969, though it has mainly focused on older people. In the case of ageism against older people, the field has used multiple definitions that have changed over time [1,283,284,285]. This potentially challenges communication about and the development of a coherent body of research on the topic. The findings from this study show that in the case of ageism towards younger persons and children, the terminology might be even more fragmented as different words have been used to describe similar concepts, thus impairing the development of a coherent body of knowledge.

The study also found that most studies come from North America or Europe and have focused on the manifestations of ageism towards younger populations, particularly in relation to employment, health and social care, power and politics, and justice. The available evidence does not enable an estimation of the global prevalence of ageism against younger people but suggests that ageism is present across institutions and prominent throughout the life course including in early life stages. It also shows that younger people might be more likely to report perceived ageism compared to other age groups.

Determinants of ageism against younger people also received a substantial amount of attention, with most studies focusing on interpersonal characteristics that may affect people’s interaction with younger people (e.g., the respondent’s personality traits). These studies, similarly to the studies that examined the manifestation of ageism towards younger people were largely a-theoretical. In fact, most of the knowledge was derived from studies that focused on ageism against older people, with younger people examined mainly as a comparison group. This can be contrasted with the literature on ageism against older persons that has attempted to explain ageism against this population group, using varied theoretical perspectives [285].

Unexpectedly, the effects of ageism against younger people have largely been understudied. This is particularly evident against the plethora of knowledge on the negative effects of ageism against older people at all levels, including the macrolevel (e.g., cost of ageism in the healthcare system [286]), and at the microlevel (e.g., negative impact of ageism on the individual’s health and wellbeing [5]). This blind spot concerning the potential impact of ageism against younger populations is particularly unfortunate and may be interpreted as yet another sign of ageism towards younger people, this time, directed by the research community, which has failed to properly examine the effects of ageism on this population group. Finally, as in the case of ageism towards older people [3], our findings show that intergenerational contact may be a useful tool to reduce ageism towards younger people. Policies might also be relevant in addressing ageism towards younger populations. However, most papers described policies without providing empirical evidence to their effect. Hence, these papers were excluded from the present review.

### 4.1. Knowledge Gaps

Several important observations emerged while reviewing the data. The first concerns the varied and somewhat inconsistent terminology used to describe ageism towards younger people. This makes the integration of the entire field complicated, as even search terms are inconsistent. Without a clear conceptual understanding of what ageism towards younger populations entails, a coherent body of knowledge cannot be formalized. In addition, many of the reviewed articles had a stated aim of examining ageism towards both younger and older adults, yet they generally focused on older adults and were derived from research and theory on ageism towards older adults. This attests to the relative research neglect of ageism towards younger people, a field that has developed mainly as a side-effect of research on older adults, with younger people serving mainly as a comparison group, and not necessarily seen as deserving research attention on their own right.

Although the manifestations of ageism towards younger people have received increasing attention over time, this attention has mostly focused on manifestations of ageism in the field of employment. It is therefore unclear whether, how, and to what extent ageism manifests in other institutional settings and sectors such as media or education, for instance. Moreover, the focus on manifestations is largely a-theoretical, thus stressing the empirical, rather than theoretical nature of research on ageism towards younger people. Indeed, there is currently no coherent theory on ageism as it affects younger populations. In addition, our review identified a paucity of research on the effects of ageism towards younger populations, which is surprising if a comparison is drawn with the abundance of evidence on the impact of this phenomenon on the health and wellbeing of older adults [5]. This study also found little published work on interventions to reduce or eliminate ageism against younger people, and the few studies that are available do not always provide a clear evaluation of the impact of the intervention, which indicates a gap for evidence-based practice, which is also apparent in research on interventions to address ageism against older people [3,285].

The limited research on children under the age of 18 is unfortunate and should be reviewed in light of the substantial reliance on college students as participants. Hence, most of our knowledge concerning ageism towards younger populations is derived from university students. Moreover, the limited research from countries outside of North America and Europe is not surprising [287], but suggests that our current knowledgebase is limited. Future research could also study the differences that may exist in experiences of ageism at different ages within the broader category of people aged 50 or below. For example, do adolescents experience ageism differently to young adults or children?

Finally, though some of the articles included in the scoping review explored how ageism can intersect with other forms of stereotyping, prejudice and discrimination, and result in cumulative disadvantage, this is an area that warrants further research. As in the case of ageism against older people, ageism against younger populations should be viewed from an intersectional perspective [166]. This is because young women, people of different ethnicities and young people of lower socioeconomic status are particularly disadvantaged in society [288]. This is illustrated by some of the evidence gathered through this scoping review which showcases the differentiated experiences of ageism when it intersects with other forms of bias.

### 4.2. Limitations

Given that this was a scoping review, no quality assessment of reviewed sources was conducted [289]. However, the review was restricted to peer-reviewed articles, whose quality was at least determined through the peer review process. It is possible that this review missed relevant articles, especially given the varied terminology used in the field. For instance, whereas terms such as youthism or kiddism are rarely used in reference to ageism towards younger people, it is possible that other research on the representations of youth might have indirectly examined ageism towards younger people. This may have been missed in our search strategy. Nevertheless, due to the inclusive search strategy and the large number of studies included, the authors believe that the current review provides a comprehensive picture of the available literature on ageism towards younger populations defined in this study as those under the age of 50. Another limitation concerns our reliance on a very crude criterion of those under 50 to identify research on ageism against younger populations. This criterion was inspired by the fact that most research on ageism against older people, and ageism more broadly, has focused on those over the age of 50 as representing the older age group [2,3,4,5]. To cover the existing knowledge gap on ageism against younger people, this study tried to identify all available evidence on ageism towards people aged 50 or below. Clearly, a more nuanced classification of individuals under the age of 50 is required to better understand ageism towards younger age groups in different contexts and settings. However, as this is the first scoping review on the topic, we decided to adopt the proposed age categorization previously used in research on ageism towards older people to develop a common and acceptable understanding of this topic. Last, this review was limited to articles in English, Spanish, and French. It is possible that research on ageism has been conducted in other languages. Future research will benefit from conducting a similar review using additional languages to better capture the manifestation of ageism in other contexts, including in low- and middle- income countries. Since the analysis for this paper was conducted, more emphasis has been given to the importance of conducting research to further explore ageism against younger populations, including in the context of the COVID-19 pandemic [290,291,292].

## 5. Conclusions

This scoping review of 263 studies covers an important gap in the research field of ageism, which has mainly focused on this phenomenon with regards to older people. It not only summarizes available evidence on ageism towards younger populations, but also highlights theoretical limitations and key research gaps, such as the limited evidence that is available on the impact of ageism towards younger people. Understanding the impact of ageism towards younger populations in the shorter term and cumulatively over the life course is key to establishing how serious a problem it is and what priority it deserves.

## Figures and Tables

**Table 1:** Main areas of study of available research on ageism against younger populations.

Main Area of Study	Num. Records	Num. Articles	% of Articles	Articles
Determinants of ageism	52	44	17%	[9,26,27,28,29,30,31,32,33,34,35,36,37,38,39,40,41,42,43,44,45,46,47,48,49,50,51,52,53,54,55,56,57,58,59,60,61,62,63,64,65,66,67,68,69]
Manifestation of ageism	267	225	86%	[6,9,28,30,31,32,33,34,35,39,40,41,42,43,44,45,46,48,49,50,51,52,53,55,56,58,59,60,61,62,64,65,66,67,69,70,71,72,73,74,75,76,77,78,79,80,81,82,83,84,85,86,87,88,89,90,91,92,93,94,95,96,97,98,99,100,101,102,103,104,105,106,107,108,109,110,111,112,113,114,115,116,117,118,119,120,121,122,123,124,125,126,127,128,129,130,131,132,133,134,135,136,137,138,139,140,141,142,143,144,145,146,147,148,149,150,151,152,153,154,155,156,157,158,159,160,161,162,163,164,165,166,167,168,169,170,171,172,173,174,175,176,177,178,179,180,181,182,183,184,185,186,187,188,189,190,191,192,193,194,195,196,197,198,199,200,201,202,203,204,205,206,207,208,209,210,211,212,213,214,215,216,217,218,219,220,221,222,223,224,225,226,227,228,229,230,231,232,233,234,235,236,237,238,239,240,241,242,243,244,245,246,247,248,249,250,251,252,253,254,255,256,257]
Effects of ageism	14	12	5%	[86,168,200,224,238,258,259,260,261,262,263,264]
Interventions to tackle ageism	34	25	9%	[88,103,106,109,112,122,264,265,266,267,268,269,270,271,272,273,274,275,276,277,278,279,280,281,282]

NOTE. Records may be higher than articles as several articles included more than one sample.

**Table 2:** Manifestations of ageism against younger populations in specific institutional settings or sectors.

	Num. Records	Num. Papers	% of Papers on Manifestations
Employment	86	75	33%
Health and social care	28	26	12%
Power and politics	16	16	7%
Justice	13	9	4%
Education	5	5	2%
Media	2	2	0.9%

## Data Availability

The data presented in this study are available in the article and supplementary material.

## Supplementary Materials

The following are available online at https://www.mdpi.com/article/10.3390/ijerph18083988/s1, Table S1: Search strategy for PubMed, Table S2: Study characteristics.
