# Supplementary material for: Scoping Review on Ageism against Younger Populations
Source: Int J Environ Res Public Health. 2021 Apr 10;18(8):3988. doi: 10.3390/ijerph18083988 (PMC8069403; doi:10.3390/ijerph18083988)
Supplement: Supplementary file 1 [file ijerph-18-03988-s001.pdf]

Scoping review on ageism against younger populations

Vânia de la Fuente-Núñez *, Ella Cohn-Schwartz, Senjooti Roy and Liat Ayalon

Supplementary Materials

S1 Table: Search strategy for PubMed

Search strategy for PubMed	("kiddism"[All fields] OR "childism"[All fields] OR "youthism"[All fields] OR "adultism"[All fields] OR "reverse ageism"[All fields] OR "reverse agism" [All fields] OR "childist"[All fields] OR "adultist"[All fields])<br>OR<br>(("child"[Mesh] OR child*[Tiab] OR "kid"[Tiab] OR "kids"[Tiab] OR "adolescent"[Mesh] OR adolesc*[Tiab] OR "girl"[Tiab] OR "girls"[Tiab] OR "boy"[Tiab] OR "boys"[Tiab] OR teen*[Tiab] OR "young adult"[Mesh] OR "young*"[Tiab] OR "middle aged"[Mesh] OR "middle age*"[Tiab] OR "mid*life"[Tiab] OR "youth*"[Tiab])<br>AND<br>("ageism"[Mesh] OR "ageism"[Tiab] OR "agism"[Tiab] OR "ageist"[Tiab] OR "agist" [Tiab] OR "age discrimination"[Tiab] OR "age prejudice"[Tiab] OR "age* stereotyp*"[Tiab] OR "age* perception*"[Tiab] OR "generation gap"[Tiab] OR "generational gap"[Tiab]))<br>NOT<br>("Animals" NOT ("Animals"[Mesh] AND "Humans"[Mesh]))<br>NOT<br>("Plants"[Mesh])

Table S2: Study characteristics

Author(s), year	Country	Main purpose of the study	Type of Research	Study design timeframe	Sampling approach	Sample size	Age [Range (Mean, SD)]	Sex (% female)	Population	Target age group studied
(Abrams, Eller, & Bryant, 2006)	United Kingdom	Determinants of ageism	Quantitative - experimental	Cross-sectional	Convenience	97	74.81(59-89,SD= 7.43)	67%	Organizations for retired people	Younger adults (<35)
(Ahammer & Baltes, 1972)	United States of America	Manifestation/incidence/prevalence/ magnitude of ageism	Quantitative - correlational	Cross-sectional	Simple Random	120	Adolescents (age 15-18), adults (34-40), and older people (64-74)	50%	Residents of an apartment complex and students in high school.	15-58, 34-40, 64-74 years old
(Ahmed, Andersson, & Hammarstedt, 2012)	Sweden	Manifestation/incidence/prevalence/ magnitude of ageism	Quantitative - experimental	Cross-sectional	Convenience	466	Not reported	Not reported	Employers	31 and 46 year olds
(Albert, Escot, & Fernández-Cornejo, 2011)	Spain	Manifestation/incidence/prevalence/ magnitude of ageism	Quantitative - experimental	Cross-sectional	Not clear	1062 job offers	Not relevant	Not relevant	Employers	Candidates aged 24, 28 or 38 years old
(Alcock, Camic, Barker, Haridi, & Raven, 2011)	United Kingdom	Interventions to tackle ageism	Qualitative - ethnography	Longitudinal	Purposive or judgement	13	65-80	77%	Residents of a housing estate	9-14 years old
(Anderson & Morgan, 2017)	United States of America	Manifestation/incidence/prevalence/ magnitude of ageism	Qualitative - not specified	Cross-sectional	Convenience	32	(9 younger: 20-34, 11, middle-aged:35-55, 12>55 years)	12.50%	Nurses	The younger generation
(Andersson, 1973)	Sweden	Manifestation/incidence/prevalence/ magnitude of ageism	Quantitative - correlational	Cross-sectional	Simple Random	87	Not reported	53%	Parents of 7th grade students	Teenagers (13 year olds) and their parents
(Andersson, 1974)	Sweden	Manifestation/incidence/prevalence/ magnitude of ageism	Quantitative - descriptive	Cross-sectional	Simple Random	50	7th grade (M=13]	50%	7th grader students	Adolescents and adults
(Andersson, 1974)	Sweden	Manifestation/incidence/prevalence/ magnitude of ageism	Quantitative - descriptive	Cross-sectional	Purposive or judgement	87	Not reported	52.80%	Parents of 7th grader students	Adolescents and adults
(Andersson, 1974)	Sweden	Manifestation/incidence/prevalence/ magnitude of ageism	Quantitative - descriptive	Cross-sectional	Not reported	181	9th grade (M=16]	52.50%	Older adolescents	Adolescents and adults
(Andersson, 1974)	Sweden	Manifestation/incidence/prevalence/ magnitude of ageism	Quantitative - descriptive	Cross-sectional	Not reported	97	12th grade [M=19]	56.70%	Older adolescents	Adolescents and adults

Author(s), year	Country	Main purpose of the study	Type of Research	Study design timeframe	Sampling approach	Sample size	Age [Range (Mean, SD)]	Sex (% female)	Population	Target age group studied
(Andersson, 1974)	Sweden	Manifestation/incidence/prevalence/ magnitude of ageism	Quantitative - descriptive	Cross-sectional	Not reported	41	18-20	100%	Female working youth	Adolescents and adults
(Andersson, 1974)	Sweden	Manifestation/incidence/prevalence/ magnitude of ageism	Quantitative - descriptive	Cross-sectional	Not reported	64	Not reported	Not reported	School personnel	Adolescents and adults
(Andersson, 1974)	Sweden	Manifestation/incidence/prevalence/ magnitude of ageism	Quantitative - descriptive	Cross-sectional	Simple Random	108	40-60	18.50%	Individuals with professions that usually require a university education	Adolescents and adults
(Andersson, 1974)	Sweden	Manifestation/incidence/prevalence/ magnitude of ageism	Quantitative - descriptive	Cross-sectional	Convenience	192	40-61	25%	People attending Bingo halls	Adolescents and adults
(Andersson, 1974)	Sweden	Manifestation/incidence/prevalence/ magnitude of ageism	Quantitative - descriptive	Cross-sectional	Not reported	86	Not reported	100%	Adolescents attending reform schools	Adolescents and adults
(Andersson, 1974)	Sweden	Manifestation/incidence/prevalence/ magnitude of ageism	Quantitative - descriptive	Cross-sectional	Not reported	97	Not reported	32.90%	Parents of adolescents attending reform schools	Adolescents and adults
(Andreoletti & Lachman, 2004)	United States of America	Consequences of ageism	Quantitative - experimental	Cross-sectional	Stratified	46	21-39 (M = 31.8; SD = 5.5)	55%	Young adults	young, middle-aged, and older adults
(Andreoletti & Lachman, 2004)	United States of America	Consequences of ageism	Quantitative - experimental	Cross-sectional	Convenience	48	21-39 (M = 31.8; SD = 5.5)	54%	College students	College students
(Andreoletti, Leszczynski, & Disch, 2015)	United States of America	Manifestation/incidence/prevalence/ magnitude of ageism	Quantitative - experimental	Cross-sectional	Convenience	134	18-25 (M=18.7, SD=1.5)	65%	University students	15, 25, 35, 45, 55, 65, 75,85, 95
(Andreoletti & Howard, 2018)	United States of America	Interventions to tackle ageism	Quantitative - experimental	Longitudinal	Convenience	21	59-101 (M=86.4, SD= 8.6)	67%	Older adults living in an assisted-living community	Young people (M=21.6 years)
(Arbuckle & Williams, 2003)	United States of America	Manifestation/incidence/prevalence/ magnitude of ageism	Quantitative - experimental	Cross-sectional	Convenience	352	17-30+	56%	University students	"young" professors (younger than 35), "old" professors (older than 55)

Author(s), year	Country	Main purpose of the study	Type of Research	Study design timeframe	Sampling approach	Sample size	Age [Range (Mean, SD)]	Sex (% female)	Population	Target age group studied
(Arslanian-Engoren, 2000)	United States of America	Manifestation/incidence/prevalence/ magnitude of ageism	Qualitative - not specified	Cross-sectional	Purposive or judgement	12	Not reported	66%	ED nurses	Not Reported
(Atkinson & Herro, 2010)	United States of America	Manifestation/incidence/prevalence/ magnitude of ageism	Qualitative - grounded theory	Longitudinal	Purposive or judgement	121	Not applicable	Not applicable	Andre Agassi	20-35
(Avolio & Barrett, 1987)	United States of America	Manifestation/incidence/prevalence/ magnitude of ageism, Determinants of ageism	Quantitative - experimental	Cross-sectional	Convenience	156	18-46 (M= 23.10, SD = 5.90)	Not clear	Students in day and evening courses	Younger and older job applicants (32, 59, no age given)
(Axt, Ebersole, & Nosek, 2014)	United States of America	Manifestation/incidence/prevalence/ magnitude of ageism	Quantitative - correlational	Cross-sectional	Not reported	49014	(M=33.3, SD = 14.1)	69%	American citizens	children, young adults, middle-age adults, older adults.
(Ayalon, 2019)	Multiple European countries (more than 20)	Manifestation/incidence/prevalence/ magnitude of ageism	Quantitative - correlational	Cross-sectional	Not reported	56170	(M=44.9, SD=18.4)	55%	Europeans over the age of 15	People in their 20s, people in their 70s
(Ayalon, 2013)	Multiple European countries (more than 20)	Determinants of ageism, Manifestation/incidence/prevalence/ magnitude of ageism	Quantitative - correlational	Cross-sectional	Simple Random	54988	Not reported	Not reported	Europeans over the age of 15	People in their 20s, people in their 70s
(Baker, 1983)	Canada	Manifestation/incidence/prevalence/ magnitude of ageism	Quantitative - experimental	Cross-sectional	Convenience	256	18-35 (M=22)	65%	University students	Ages 5, 10, 20, 30, 50, 70, and 90
(Banjare, Pradhan, Dwivedi, Mahapatra, & Debata, 2017)	India	Manifestation/incidence/prevalence/ magnitude of ageism	Quantitative - correlational	Cross-sectional	Systematic Random	310	60+	51%	Older adults aged 60+	Young people
(Banziger & Drevenstedt, 1982)	United States of America	Manifestation/incidence/prevalence/ magnitude of ageism	Quantitative - experimental	Cross-sectional	Convenience	176	M=18.58, SD=1.5	100%	University students	30 vs. 70
(Banziger & Drevenstedt, 1982)	United States of America	Manifestation/incidence/prevalence/ magnitude of ageism	Quantitative - experimental	Cross-sectional	Convenience	176 Undergraduate women, 96 older women	Undergraduate women: M=19.23, SD=1.86; Older women: M=73, 55-82	100%	University women and older women	30 vs. 70

Author(s), year	Country	Main purpose of the study	Type of Research	Study design timeframe	Sampling approach	Sample size	Age [Range (Mean, SD)]	Sex (% female)	Population	Target age group studied
(Barnes-Farrell & Ross, 1992)	United States of America	Manifestation/incidence/prevalence/ magnitude of ageism	Quantitative - experimental	Cross-sectional	Convenience	84	(Mean=32.3)	41%	University students and employees from a manufacturing plant	younger (mean perceived age=32.1) or older (mean perceived age=58.6).
(Bastos, Barros, Celeste, Paradies, & Faerstein, 2014)	Brazil	Manifestation/incidence/prevalence/ magnitude of ageism, Consequences of ageism	Quantitative - correlational	Cross-sectional	Not clear	424	18-35	59%	Brazilian university students	18-35 years old
(Belgrave, 2011)	United States of America	Interventions to tackle ageism	Quantitative - experimental	Longitudinal	Convenience	26 older adults (21 children)	Not reported	Not reported	Older adults from a retirement living facility (+4th grade class)	4th grade children
(Bengtson, 1971)	United States of America	Manifestation/incidence/prevalence/ magnitude of ageism	Quantitative - descriptive	Cross-sectional	Simple Random	278	Not reported	Not reported	Members of three-generation families	15-26 years and their grandparents
(Bennington, 2001)	Australia	Manifestation/incidence/prevalence/ magnitude of ageism, Interventions to tackle ageism	Qualitative - not specified	Cross-sectional	Purposive or judgement	180 job advertisements	Not relevant	Not relevant	Positions for nonspecialized (e.g. nonmedical and nonlegal) secretarial positions.	under 20s, early 20s, late 30s, over 50s
(Bennington, 2001)	Australia	Manifestation/incidence/prevalence/ magnitude of ageism, Interventions to tackle ageism	Quantitative - experimental	Cross-sectional	Purposive or judgement	180 job advertisements	Not relevant	Not relevant	Positions for nonspecialized (e.g. nonmedical and nonlegal) secretarial positions.	one of the three ages (23, 37, or 51 years)
(Bennington, 2001)	Australia	Manifestation/incidence/prevalence/ magnitude of ageism, Interventions to tackle ageism	Qualitative - not specified	Cross-sectional	Simple Random	180	Not reported	35%	Employers	25 and above
(Bennington, 2001)	Australia	Manifestation/incidence/prevalence/ magnitude of ageism, Interventions to tackle ageism	Qualitative - not specified	Cross-sectional	Purposive or judgement	186	18-61, M=30	Not reported	Job applicants	18-61
(Bensimon & Bodner, 2012)	Israel	Manifestation/incidence/prevalence/ magnitude of ageism	Quantitative - experimental	Cross-sectional	Convenience	129	20-42 (M=24.69, SD=4.26)	77%	University students	20-40 years old vs 70-80 year olds
(Bergland, Nicolaisen, & Thorsen, 2014)	Norway	Determinants of ageism	Quantitative - correlational	Longitudinal	Stratified, Systematic Random	2471	40-79	52.40%	Adults	Age groups of participants

Author(s), year	Country	Main purpose of the study	Type of Research	Study design timeframe	Sampling approach	Sample size	Age [Range (Mean, SD)]	Sex (% female)	Population	Target age group studied
(Bertolino, Truxillo, & Fraccaroli, 2013)	Italy	Manifestation/incidence/prevalence/ magnitude of ageism, Determinants of ageism	Quantitative - experimental	Cross-sectional	Not clear	155	25-61 (M=44.70, SD=7.47)	84%	Clerical and financial administration employees in schools.	24-34 year old; 55-65 year old
(Blatt-Eisengart & Lachman, 2004)	United States of America	Manifestation/incidence/prevalence/ magnitude of ageism	Quantitative - experimental	Cross-sectional	Simple Random	149 including 50 young adults, 50 middle-aged adults, and 49 older adults	Young adults: 21-40 (M=32.4, SD=5.7). (Middle-aged adults: 41-60 (M=48.5, SD=5.4). Older adults: 61-80 (M=69.6, SD=5.3))	65%	Adults	Younger adults (middle-aged adults and older adults)
(Boduroglu, Yoon, Luo, & Park, 2006)	United States of America, China	Manifestation/incidence/prevalence/ magnitude of ageism	Quantitative - descriptive	Cross-sectional	Convenience	80	Younger Americans (M=18.91, SD=0.73); Younger Chinese (M=21.22, SD= 1.31); Older Americans (M=7.10, SD=4.35); Older Chinese (M=66.16, SD=1.86)	50%	University students; the older Americans were community-dwelling and the older Chinese participants were recruited from a pool of retired staff and faculty at the National Academy of Sciences.	Young people; Older people
(Boyd & Dowd, 1988)	United States of America	Manifestation/incidence/prevalence/ magnitude of ageism	Quantitative - experimental	Cross-sectional	Convenience	20	18-47	Not reported	Students, faculty and staff of the university	Young, middle age, old
(Bratt, Abrams, Swift, Vauclair, & Marques, 2018)	Multiple European countries (more than 20)	Manifestation/incidence/prevalence/ magnitude of ageism	Quantitative - correlational	Cross-sectional	Simple Random	56272	15-105 (M=47.54, SD=18.5)	48%	Europeans over the age of 15	Young people, older people
(Brewer & Lui, 1989)	United States of America	Manifestation/incidence/prevalence/ magnitude of ageism	Quantitative - descriptive	Cross-sectional	Convenience	60	Not reported	approx. 50%	University students	Younger (between 21 and 40) and older adults' (between 55 and 90) photographs
(Brewer & Lui, 1989)	United States of America	Manifestation/incidence/prevalence/ magnitude of ageism	Quantitative - descriptive	Cross-sectional	Not reported	58	Not reported	Not reported	Not reported	Younger (between 21 and 40) and older adults' (between 55 and 90) photographs

Author(s), year	Country	Main purpose of the study	Type of Research	Study design timeframe	Sampling approach	Sample size	Age [Range (Mean, SD)]	Sex (% female)	Population	Target age group studied
(Cai, Giles, & Noels, 1998)	China	Manifestation/incidence/prevalence/ magnitude of ageism	Quantitative - correlational	Cross-sectional	Not reported	197	48-86 (M=64.06)	44.70%	Han Chinese adults	Young non-family adults; young family adults, older non-family adults
(Callan, Dawtry, & Olson, 2012)	United Kingdom	Manifestation/incidence/prevalence/ magnitude of ageism	Quantitative - experimental	Cross-sectional	Convenience	53	(M=30.25, SD=14.53)	47%	Art gallery participants	18 year old, 74 year old
(Callan et al., 2012)	United Kingdom	Manifestation/incidence/prevalence/ magnitude of ageism	Quantitative - experimental	Cross-sectional	Convenience	119	(M=26.68, SD=8,91)	45%	University students and staff	14 year old, 84 year old
(Callan et al., 2012)	United Kingdom	Manifestation/incidence/prevalence/ magnitude of ageism	Quantitative - experimental	Cross-sectional	Convenience	120	(M=23.10, SD=8.48)	52%	People around the University campus	12 year old, 82 year old
(Cameron, 1970b)	United States of America	Manifestation/incidence/prevalence/ magnitude of ageism	Quantitative - correlational	Cross-sectional	Not reported	317	18-25; 40-55; 65-79	50%	An area sample of white Detroiters	18-25, 40-55 and 65-79
(Cameron, 1973)	United States of America	Manifestation/incidence/prevalence/ magnitude of ageism	Quantitative - descriptive	Cross-sectional	Convenience	317	Evenly divided as young, middle-aged, and old	50%	Not reported	Own generation and others' generation (18-25, 40-55, 65-79)
(Cameron, 1970a)	United States of America	Manifestation/incidence/prevalence/ magnitude of ageism	Quantitative - descriptive	Cross-sectional	Convenience	317	young adults were aged 18 to 25 inclusive (M=21), middle-aged from 40 to 55 (M=48.2), and old from 65 to 79 (M=70.2)	Not reported	Residents of city of Detroit (excluding the inner city), Caucasians only	aged 18 to 25, aged 40-55, aged 65-79
(Cary, Chasteen, & Cadieux, 2013)	Canada	Determinants of ageism, Manifestation/incidence/prevalence/ magnitude of ageism	Quantitative - experimental	Cross-sectional	Convenience	109	Young adults: 18-23 (M = 18.73) Older adults: 60-80 (M= 70.48) / 18-80	52.5% (young adults); 50% (older adults)	University students and seniors	Young adults (18-23), older adults (60-80)
(Casper, Rothermund, & Wentura, 2011)	Germany	Determinants of ageism	Quantitative - experimental	Cross-sectional	Convenience	56	Not reported	Not reported	University students	Young people
(Casper et al., 2011)	Germany	Determinants of ageism	Quantitative - experimental	Cross-sectional	Convenience	48	Not reported	Not reported	University students	Young people
(Casper et al., 2011)	Germany	Determinants of ageism	Quantitative - experimental	Cross-sectional	Convenience	38	Not reported	Not reported	University students	Young people
(Casper et al., 2011)	Germany	Determinants of ageism	Quantitative - experimental	Cross-sectional	Convenience	38	Not reported	Not reported	University students	Young people

**Author(s), year**	**Country**	**Main purpose of the study**	**Type of Research**	**Study design timeframe**	**Sampling approach**	**Sample size**	**Age [Range (Mean, SD)]**	**Sex (% female)**	**Population**	**Target age group studied**
(Ceaser, 2014)	United States of America	Manifestation/incidence/prevalence/ magnitude of ageism	Qualitative - ethnography	Cross-sectional	Purposive or judgement	Groups of 10-20 students and a group of 5-10 local youth; 5 staff members; 3 other adults (volunteer, farm owner and founder's friend)	16-21 years (students) 5-15 (local youth) +8 adults	Approx. 50% students; sdults not reported	College and high school students	16-21 years
(Chan et al., 2012)	Multiple countries (more than 20)	Manifestation/incidence/prevalence/ magnitude of ageism, Measurement of ageism	Quantitative - correlational	Cross-sectional	Not reported	3323	Reported per country. On average participants were in their early 20s	Reported per country. On average, two-thirds were female	Native born citizens	Adolescents (perceived to start at a median age of 13 and to end at a median age of 19 years of age; adulthood perceived to start at age 21 and to end at age 59; and old age perceived to start at age 60.
(Chen & King, 2002)	United States of America	Manifestation/incidence/prevalence/ magnitude of ageism	Quantitative - correlational	Cross-sectional	Convenience	118 - young adults; 102 older adults / 220	18-29 (M=21.68, SD=3.18); 60-90 (M=73.00, SD=6.64)	Not reported	University students and older volunteers from community organizations	20-year-old woman, 70-year-old woman
(Chen, Pethtel, & Ma, 2010)	United States of America	Interventions to tackle ageism	Quantitative - experimental	Cross-sectional	Convenience	129	18-24 (M=19.19, SD=1.19); 61-89 (M=77.18, SD=6.79)	67%	University students and older adults	23 years old, 70 years old
(Chien & Tann, 2017)	China	Interventions to tackle ageism	Quantitative - experimental	Longitudinal	Convenience	196	51-85 (M=65.77, SD=9.114); 20-21 (M= 20.4, SD=0.516); 7-13 (M=9.28, SD=1.224)	85%	Older participants from community senior centers and elementary schools students	7-13; 20-21
(Chu & Grühn, 2018)	United States of America	Manifestation/incidence/prevalence/ magnitude of ageism	Quantitative - experimental	Cross-sectional	Convenience	563	18–72 (M=29.86, SD=12.12)	43%	College students and adults from Amazon Mechanical Turk	young, old victims and perpetrators
(Chu & Grühn, 2018)	United States of America	Manifestation/incidence/prevalence/ magnitude of ageism	Quantitative - experimental	Cross-sectional	Convenience	509	18–71 (M=30.94, SD=12.51)	Not reported	College students and adults from Amazon Mechanical Turk	young, old victims and perpetrators

Author(s), year	Country	Main purpose of the study	Type of Research	Study design timeframe	Sampling approach	Sample size	Age [Range (Mean, SD)]	Sex (% female)	Population	Target age group studied
(Chua & Theng, 2013)	Singapore	Interventions to tackle ageism	Quantitative - experimental	Longitudinal	Convenience	19 youth+19 older adults- video game; 18 youth+18 older adults nonvideo game	Videogame Youth: 16-18 (M=16.74, SD=0.65); Videogame older adults: 60-86 (M=75.42, SD=8.15); Nonvideo game Youth: 16-19 (M=17.67, SD=0.84); Nonvideo game older adults: 60-89 (M= 76.5, SD=7.33)	Videogame youth: 63%; Videogame older adults: 95%; Nonvideo game youth: 72% Nonvideo game older adults: 83%	College students and older adults from senior activity centers	16-19 year olds vs 60-89 year olds
(Cleveland & Landy, 1981)	United States of America	Manifestation/incidence/prevalence/ magnitude of ageism, Determinants of ageism	Quantitative - descriptive	Cross-sectional	Convenience	150 raters (rating 513 ratees aged 21-65)	Not reported	0%	Supervisors of managers	younger workers (21-34), middle-age (35-44), older (45-65)
(Cleveland & Landy, 1981)	United States of America	Manifestation/incidence/prevalence/ magnitude of ageism, Determinants of ageism	Quantitative - descriptive	Cross-sectional	Convenience	Number of raters not reported (rating 178 ratees aged 22-64)	Not reported	Not reported	Supervisors of employees	younger workers (21-34), middle-age (35-44), older (45-65)
(Coleman, George, & Holt, 1977)	United Kingdom	Manifestation/incidence/prevalence/ magnitude of ageism	Quantitative - correlational	Cross-sectional	Convenience	48	Preadolescent: M=10 years 02 months; Adolescent: M=15 years 05 months; mothers' ages not reported	50%	Primary school students, youth and mothers	Average teenager, average adult
(Conner, 2016)	United States of America	Manifestation/incidence/prevalence/ magnitude of ageism	Qualitative - case study	Cross-sectional	Snowball	22	Not reported	27%	Current and former youth commissioners and adults who represented the target audience for BCYC's work or who partnered with the commission on various initiatives	Youth
(Conner, Ober, & Brown, 2016)	United States of America	Manifestation/incidence/prevalence/ magnitude of ageism	Qualitative - case study	Cross-sectional	Purposive or judgement	31	32-65	Not reported	Individuals who were deeply involved in education policy	youth organizers

Author(s), year	Country	Main purpose of the study	Type of Research	Study design timeframe	Sampling approach	Sample size	Age [Range (Mean, SD)]	Sex (% female)	Population	Target age group studied
(Cortellesi & Kernan, 2016)	Italy, Ireland, Netherlands, Poland, Portugal, Spain, Slovenia	Interventions to tackle ageism	Qualitative - case study	Cross-sectional	Convenience	500 children; 303 older adults; 111 facilitators	0-8 children, 65+ older adults	Not reported	Case study- 2-3 cases per country that reflected intergenerational programs	0-8
(Cortellesi & Kernan, 2016)	Italy, Ireland, Netherlands, Poland, Portugal, Spain, Slovenia	Interventions to tackle ageism	Qualitative - not specified	Cross-sectional	Convenience	589 children; 163 older adults; 101 facilitators	0-8 children, 65+ older adults	Not reported	13 pilot intergenerational programs in 5 countries	0-8
(Cullen, Barnes-Holmes, Barnes-Holmes, & Stewart, 2009)	Ireland	Manifestation/incidence/prevalence/ magnitude of ageism, Measurement of ageism	Quantitative - experimental	Cross-sectional	Not reported	12	18 - 26 (M = 21)	58%	University students	Young people, old people
(Cullen et al., 2009)	Ireland	Determinants of ageism, Manifestation/incidence/prevalence/ magnitude of ageism, Measurement of ageism	Quantitative - experimental	Longitudinal	Not reported	24	18 - 26 (M = 19)	67%	Not reported	Young people, old people
(Cunha, Marques, & Borges Rodrigues, 2014)	Portugal	Manifestation/incidence/prevalence/ magnitude of ageism	Quantitative - descriptive	Cross-sectional	Convenience	69	11-16; 52 -82	Not reported	Participants in the institution	Young people
(Cunha et al., 2014)	Portugal	Interventions to tackle ageism	Qualitative - content analysis	Longitudinal	Purposive or judgement	5	52-72	Not reported	Participants in the activity	Young people (aged 13-15)
(DeArmond et al., 2006)	United States of America	Manifestation/incidence/prevalence/ magnitude of ageism	Quantitative - experimental	Cross-sectional	Convenience	496	(M=18.8, SD=1.25)	61.90%	University students	25, 40, or 55 year olds
(Dedrick & Dobbins, 1991)	United States of America	Manifestation/incidence/prevalence/ magnitude of ageism	Quantitative - experimental	Cross-sectional	Convenience	124	Not reported	Not reported	University students	30 years old worker vs 60 year old
(DeSouza, 2007)	Brazil	Manifestation/incidence/prevalence/ magnitude of ageism, Interventions to tackle ageism, Theory development	Qualitative - not specified	Cross-sectional	Simple Random	32	60+	Not reported	Participants in the activity	Students aged 12-18

Author(s), year	Country	Main purpose of the study	Type of Research	Study design timeframe	Sampling approach	Sample size	Age [Range (Mean, SD)]	Sex (% female)	Population	Target age group studied
(Diekman & Hirnisey, 2007)	United States of America	Determinants of ageism, Manifestation/incidence/prevalence/ magnitude of ageism	Quantitative - experimental	Cross-sectional	Convenience	41	Median=19	56%	University students	Young candidate vs older candidate
(Diekman & Hirnisey, 2007)	United States of America	Determinants of ageism, Manifestation/incidence/prevalence/ magnitude of ageism	Quantitative - experimental	Cross-sectional	Convenience	209	Median=19	62%	University students	35 years old, 65 years old
(Diekman & Hirnisey, 2007)	United States of America	Determinants of ageism, Manifestation/incidence/prevalence/ magnitude of ageism	Quantitative - experimental	Cross-sectional	Convenience	108	Median=19	56%	University students	Young candidate vs older candidate
(Dillard & Coupland, 1990)	United Kingdom	Manifestation/incidence/prevalence/ magnitude of ageism	Quantitative - experimental	Cross-sectional	Convenience	134	(M=18.75, SD=2.5)	63.40%	College students	young person, 21 year old, 70-71 year old
(Dioux, Brochard, Gabarrot, & Zagar, 2016)	France	Manifestation/incidence/prevalence/ magnitude of ageism	Quantitative - experimental	Cross-sectional	Convenience	81	19-25 (M= 21.02)	93%	University students	Young people, older people
(Dioux et al., 2016)	France	Manifestation/incidence/prevalence/ magnitude of ageism	Quantitative - experimental	Cross-sectional	Convenience	144	(M= 20.5)	82%	University students	Young people, older people
(Döbrich & Spörrle, 2014)	United States of America	Interventions to tackle ageism, Manifestation/incidence/prevalence/ magnitude of ageism	Quantitative - experimental	Cross-sectional	Convenience	176	22-77 (M = 42.18, SD = 11.00)	50%	HR professionals	28 year and 59 year old candidate
(Döbrich & Spörrle, 2014)	United States of America	Interventions to tackle ageism	Quantitative - experimental	Cross-sectional	Convenience	384	20 - 75 (M = 36.16, SD = 10.99)	50%	HR professionals, managers, headhunters, consultants, and retirees who formerly belonged to one of these professional groups.	young and old worker
(Doubleday & Lee, 2016)	United States of America	Manifestation/incidence/prevalence/ magnitude of ageism	Quantitative - experimental	Cross-sectional	Convenience	131	M=23.5	Not clear	Dental students	Younger people (22–25 years old), older people (late 60s)
(Douthirt-Cohen & Tokunaga, 2019)	Japan, United States of America	Manifestation/incidence/prevalence/ magnitude of ageism	Qualitative - ethnography	Longitudinal	Purposive or judgement	2	Late 20's 30's	100%	2 researchers reflecting on their research	15-21; 14-18; 13-17
(Dow, Joosten, Biggs, & Kimberley, 2016)	Australia	Manifestation/incidence/prevalence/ magnitude of ageism, Interventions to tackle ageism	Qualitative - content analysis	Cross-sectional	Convenience	20	16–23 (M=19.5); 65–89 (M=79.75)	55%	Older and young adults	Younger people (16-23), older people (65-89)

Author(s), year	Country	Main purpose of the study	Type of Research	Study design timeframe	Sampling approach	Sample size	Age [Range (Mean, SD)]	Sex (% female)	Population	Target age group studied
(Doyle, Bottomley, & Angell, 2017)	United Kingdom	Measurement of ageism	Quantitative - correlational	Longitudinal	Not clear	Not applicable	Not applicable	Not applicable	Not applicable	Younger people in an age-banded cohort, typically a school- or competition-year.
(Drehmer, Carlucci, Bordieri, & Pincus, 1992)	United States of America	Manifestation/incidence/prevalence/ magnitude of ageism	Quantitative - experimental	Cross-sectional	Convenience	56	Median=26	Not reported	Supervisors and middle-managers	Ages 25, 29, 31, 43. Also 52, 59 and 63
(Drydakis & Somers, 2018)	United Kingdom	Manifestation/incidence/prevalence/ magnitude of ageism	Quantitative - experimental	Cross-sectional	Not clear	894 pairs of matched job applications differing only in age of applicant (28 year old or 50 year old)	Not reported	Not reported	Recruitment offices	28 year-old, 50 year-old applicant
(Duncan & Loretto, 2004)	United Kingdom	Manifestation/incidence/prevalence/ magnitude of ageism	Quantitative - correlational	Cross-sectional	Convenience	1128	16-44	54%	Employees	Younger employees, older employees
(Einarsdóttir, Jónína, & Guðbjörg, 2015)	Iceland	Manifestation/incidence/prevalence/ magnitude of ageism	Quantitative - correlational	Cross-sectional	Simple Random	952	13-17	Not reported	Icelanders aged 13-17	13-17
(Einarsdóttir et al., 2015)	Iceland	Manifestation/incidence/prevalence/ magnitude of ageism	Qualitative - not specified	Cross-sectional	Convenience, Snowball	42	13-17	Not reported	Residents of Iceland who had at least some experience of paid work.	13-17
(Erber, Szuchman, & Etheart, 1993)	United States of America	Manifestation/incidence/prevalence/ magnitude of ageism	Quantitative - experimental	Cross-sectional	Convenience	179	17-35 (M=22.8)	49%	Young adults and university students	32 year-old, 64 year-old
(Erber et al., 1993)	United States of America	Manifestation/incidence/prevalence/ magnitude of ageism	Quantitative - experimental	Cross-sectional	Convenience	90	18-35 (M=23.0)	56.60%	Young adults and university students	33 year-old, 64 year-old
(Erber & Szuchman, 2002)	United States of America	Manifestation/incidence/prevalence/ magnitude of ageism	Quantitative - experimental	Cross-sectional	Convenience	139	18-75 (M=35.26, SD=14.32)	Approx. 63%	Young adults and university students	28 or 67 years of age
(Erber & Long, 2006)	United States of America	Manifestation/incidence/prevalence/ magnitude of ageism	Quantitative - experimental	Cross-sectional	Convenience	167	19-43 (M=24.77, SD=4.99)	63%	People who attended evening classes at a university	28 (31) year olds (younger targets) and 61 (55) year olds (older targets)

Author(s), year	Country	Main purpose of the study	Type of Research	Study design timeframe	Sampling approach	Sample size	Age [Range (Mean, SD)]	Sex (% female)	Population	Target age group studied
(Erber & Danker, 1995)	United States of America	Manifestation/incidence/prevalence/ magnitude of ageism, Determinants of ageism	Quantitative - experimental	Cross-sectional	Convenience	128	19-55 (M= 34.43, SD = 8.12)	52%	Employees	32 year old candidate, 62 year old candidate
(Erber, Szuchman, & Prager, 2001)	United States of America	Determinants of ageism, Manifestation/incidence/prevalence/ magnitude of ageism	Quantitative - experimental	Cross-sectional	Convenience	Young: 72; old: 77	Young: 18-33, M=20.63; old: 60-93, M=74.2	Young=73%; old=60%	University students and old-community living adults	20s vs 70s
(Erber et al., 2001)	United States of America	Determinants of ageism, Manifestation/incidence/prevalence/ magnitude of ageism	Quantitative - experimental	Cross-sectional	Convenience	Young: 106; old:93	Young: 19-35, M=23.28; old: 62-90, M=72.44	Young=67%; old=67%	University students and old-community living adults	20s vs 70s
(Fabes & Martin, 1991)	United States of America	Manifestation/incidence/prevalence/ magnitude of ageism	Quantitative - experimental	Cross-sectional	Convenience	400	17-51 (M=20.54)	50%	College students	infants (younfer than 2 yrs), preschoolers (3-5 yrs), elementary schoolers (6-10 yrs), adolescents (13-16), and adults (21+)
(Farney & Breault, 2006)	United States of America	Manifestation/incidence/prevalence/ magnitude of ageism, Determinants of ageism	Quantitative - correlational	Cross-sectional	Convenience	291	18-90	69%	University students, middle-aged adults and older adults from senior centers	Not relevant
(Fenwick, Cullen, Gamble, & Sidebotham, 2016)	Australia	Manifestation/incidence/prevalence/ magnitude of ageism	Qualitative - not specified	Cross-sectional	Purposive or judgement	11	17-20	Not reported	Midwifery students	Young midwifes
(Finkelstein, Voyles, Thomas, & Zacher, 2019)	United States of America	Manifestation/incidence/prevalence/ magnitude of ageism, Interventions to tackle ageism	Quantitative - descriptive	Longitudinal	Not clear	185	25-63 (M=44.35, SD=10.15)	49.70%	Employees	Ages between 25-63
(Finkelstein, Ryan, & King, 2013)	United States of America	Manifestation/incidence/prevalence/ magnitude of ageism	Qualitative - not specified	Cross-sectional	Convenience	247: 125 young; 61 middle-aged; 61 older.	Young M=21, SD=2.07, 18-29; middle-age M=46.38 SD=4.95, 33-50; old M=57.52 SD=6.82, 51-84	Young 81%; middle age 65%; old 70%	University students, middle-aged and older adults	Young, middle age, older
(Finkelstein, Higgins, & Clancy, 2000)	United States of America	Manifestation/incidence/prevalence/ magnitude of ageism	Quantitative - experimental	Cross-sectional	Convenience	324	M41.1, SD=8.6, 23-69	32.40%	Managers	28 vs 59
(Finkelstein & Burke, 1998)	United States of America	Manifestation/incidence/prevalence/ magnitude of ageism	Quantitative - experimental	Cross-sectional	Convenience	324	23-69 (M=41.1, SD=8.6)	32.70%	Managers	28 years vs 59

Author(s), year	Country	Main purpose of the study	Type of Research	Study design timeframe	Sampling approach	Sample size	Age [Range (Mean, SD)]	Sex (% female)	Population	Target age group studied
(Forte & Hansvick, 1999)	United States of America	Manifestation/incidence/prevalence/ magnitude of ageism	Quantitative - experimental	Cross-sectional	Simple Random	98	21-68 (M=44.6)	36%	Employers	worker 49 years of age or younger; worker 50 or above
(Furnham, Ariffin, & McClelland, 2007)	United States of America	Manifestation/incidence/prevalence/ magnitude of ageism	Quantitative - experimental	Cross-sectional	Convenience	523	86% were between 19-22 years old; rest were 32+	41%	University students	25 year old, 40-year old, 55 year-old
(Furnham & Briggs, 1993)	United Kingdom	Manifestation/incidence/prevalence/ magnitude of ageism	Quantitative - experimental	Cross-sectional	Convenience	100	M=24.02, 18-55	50%	Professionals and students	25-28 (young) vs. 41-44(old)
(Fusilier & Hitt, 1983)	United Kingdom	Manifestation/incidence/prevalence/ magnitude of ageism	Quantitative - correlational	Cross-sectional	Convenience	155	15-82	72%	University students and patients at a general practice surgery.	20 year olds, 50 year olds
(Garstka, Schmitt, Branscombe, & Hummert, 2004)	United States of America	Consequences of ageism	Quantitative - correlational	Cross-sectional	Convenience	59	17-20 (M=18.8)	52% (expected but not examined)	University students	young adults (17-25), older adults (over 64 years)
(Garstka, Hummert, & Branscombe, 2005)	United States of America	Manifestation/incidence/prevalence/ magnitude of ageism	Quantitative - experimental	Cross-sectional	Convenience	161	Young adults: 18-26 (M=21.0) Middle-aged adults: 33-50 (M=41.7) Older adults: 61-92 (M= 77.2)	Young adults: 50% Middle-aged adults: 60% Older adults: 66%	University students, middle-aged and older adults	Young Adults, middle-aged, older adults
(Gasson & Julie, 2015)	New Zealand	Manifestation/incidence/prevalence/ magnitude of ageism	Qualitative - not specified	Cross-sectional	Purposive or judgement	9	41-50	89%	Parents aged 40–50 years, with one or more children between the ages of 11 and 15 years	children between the ages of 11 and 15 years

Author(s), year	Country	Main purpose of the study	Type of Research	Study design timeframe	Sampling approach	Sample size	Age [Range (Mean, SD)]	Sex (% female)	Population	Target age group studied
(Gee & Long, 2007)	United States of America	Manifestation/incidence/prevalence/ magnitude of ageism	Quantitative - correlational	Longitudinal	Systematic Random	Birth cohort of 1922-1926: 952-515; Birth cohort of 1927-1931: 948-642; Birth cohort of 1932-1936: 886-709; Birth cohort of 1943-1947: 603-1116; Birth cohort of 1948-1952: 485-1460	Birth cohort 1922-1926: M47.9-64.8; Birth cohort 1927-1931: M43.1-60.1;Birth cohort 1932-1933: M38.0-54.9; Birth cohort 1943-1947: M26.7-42.7; Birth cohort 1948-1952: M22.6-37.9	100%- somewhat unclear	Women in the workforce	all ages in the workforce
(Gekoski & Knox, 1990)	United States of America	Determinants of ageism, Manifestation/incidence/prevalence/ magnitude of ageism	Quantitative - experimental	Cross-sectional	Convenience	160	18-24 (M=20.4)	50%	University students	Young, old
(Gewirtz-Meydan & Ayalon, 2017)	Israel	Manifestation/incidence/prevalence/ magnitude of ageism	Quantitative - experimental	Cross-sectional	Convenience	236	Not reported	48.70%	Israeli physicians	A 28 year old and a 78 year old
(Gibson & Franken, 1993)	Canada	Manifestation/incidence/prevalence/ magnitude of ageism, Measurement of ageism	Quantitative - correlational	Cross-sectional	Not clear	811	Not reported	Not reported	Hiring personnel	older, younger worker
(Giles, Liang, Noels, & McCann, 2001)	United States of America, China	Manifestation/incidence/prevalence/ magnitude of ageism	Quantitative - descriptive	Cross-sectional	Convenience	203 (47 Euromerican, 60 Chinese American, 100 Taiwan	17-28	70% for Euromerican; 55% Chinese American; 55% Taiwanese	University students	Same age 17-35 vs. older 65 and older
(Giles et al., 2000)	United States of America, Canada, Australia, New Zealand, China, Japan, Philippines, India, Singapore	Manifestation/incidence/prevalence/ magnitude of ageism	Quantitative - correlational	Cross-sectional	Convenience	1409	(M=19.98, SD=2.19)	52%	University students	young, middle-aged, and older adults

Author(s), year	Country	Main purpose of the study	Type of Research	Study design timeframe	Sampling approach	Sample size	Age [Range (Mean, SD)]	Sex (% female)	Population	Target age group studied
(Giles, Ryan, & Anas, 2008)	Canada	Manifestation/incidence/prevalence/ magnitude of ageism	Quantitative - correlational	Cross-sectional	Simple Random, Convenience	240	Young adults: 17-22 (M=19, SD=1.1), Middle-aged adults: 40-58 (M=48.4, SD=5.1), Older adults: 64-94 (M=75.3, SD=6.0).	Young adults: 52.5% Middle-aged adults:51.2% Older adults: 51.21%	University students, community dwelling middle-aged and older adults	17-30 years, 31-64, 65+
(Giles & Williams, 1994)	United States of America	Manifestation/incidence/prevalence/ magnitude of ageism	Qualitative - content analysis	Cross-sectional	Convenience	123	(M=18.5, SD=0.99)	72%	University students	Younger people (M=18.5)
(Giles & Williams, 1994)	United States of America	Manifestation/incidence/prevalence/ magnitude of ageism	Quantitative - experimental	Cross-sectional	Convenience	252	(M=19.3, SD=1.47)	56%	University students	20 years-old
(Gluth, Ebner, & Schmiedek, 2010)	Germany	Measurement of ageism, Determinants of ageism,	Quantitative - correlational	Cross-sectional	Convenience	151 (younger participants); 143 (older participants)	Younger participants: 18-31 (M=24.8, SD=3.1) Older participants: 68-81 (M=73.4, SD= 3.1)	Younger participants: 51%. Older participants: 46.9%	Younger and older adults from the community	Older adults and younger adults (no age specified)
(Goebel, 1984)	United States of America	Manifestation/incidence/prevalence/ magnitude of ageism	Quantitative - correlational	Cross-sectional	Convenience	72	18-48 (M = 21.6)	100%	Caucasian female nursing students	children, adolescents, young adults, middle-aged adults, and old adults
(Goebel & Cashen, 1985)	United States of America	Determinants of ageism	Quantitative - experimental	Cross-sectional	Convenience	168	Not reported	Not reported	University students	Young, middle aged, old
(Goldberg & Shore, 2003)	United States of America	Manifestation/incidence/prevalence/ magnitude of ageism	Quantitative - correlational	Cross-sectional	Convenience	304-311	Recruiters: (M=37.5, SD=8.29) Applicants: (M=27.5, SD=6.04)	Recruiters: 33.33% Applicants: 34.3%	Applicants and recruiters in colleges	young and middle aged job seekers
(H. R. Gordon, 2007)	United States of America	Manifestation/incidence/prevalence/ magnitude of ageism	Qualitative - ethnography	Cross-sectional	Purposive or judgement	40	Not reported	Not reported	Two youth movement organizations	Youth and young adult activists
(R. A. Gordon, Rozelle, & Baxter, 1988)	United States of America	Manifestation/incidence/prevalence/ magnitude of ageism	Quantitative - correlational	Cross-sectional	Convenience	150	18-87 (M=35.4, SD=15.3)	61.30%	University students, office personnel, churchgoers, and respondents from a senior citizens' group.	Full spectrum of ages

Author(s), year	Country	Main purpose of the study	Type of Research	Study design timeframe	Sampling approach	Sample size	Age [Range (Mean, SD)]	Sex (% female)	Population	Target age group studied
(R. A. Gordon, Rozelle, & Baxter, 1989)	United States of America	Determinants of ageism	Quantitative - experimental	Cross-sectional	Convenience	120	18-68 (M=24.75)	60%	University students	25, 40, 55 year olds
(R. A. Gordon & Arvey, 1986)	United States of America	Determinants of ageism	Quantitative - experimental	Cross-sectional	Convenience	120	18-56 (M=25.87)	50%	University students	25 and 40 years old, or 55 year old
(Graham & Baker, 1989)	United States of America	Manifestation/incidence/prevalence/ magnitude of ageism	Quantitative - experimental	Cross-sectional	Snowball	198	45-92 (M=67)	Not clear	Older adults from seniors associations and organizations	ages 5, 20, 30, 40, 50, 65, 70, 80 and 100
(Granleese & Sayer, 2006)	United States of America	Manifestation/incidence/prevalence/ magnitude of ageism	Qualitative - phenomenolo gy	Cross-sectional	Purposive or judgement	48	<30-33%; 30-45-33%; >45-33%	50%	Academics and non-academics who work in higher education	Young – under 30 years old; Middle – 30-45 years old
(Gross & Hardin, 2007)	United States of America	Manifestation/incidence/prevalence/ magnitude of ageism	Quantitative - experimental	Cross-sectional	Convenience	106	(M=21.5, SD=4.2)	79%	University students	Adolescents
(Gross & Hardin, 2007)	United States of America	Manifestation/incidence/prevalence/ magnitude of ageism, Determinants of ageism	Quantitative - experimental	Cross-sectional	Convenience	37	(M=20.8, SD=2.5)	51%	University students	Adolescents
(Haber, 1970)	United States of America	Manifestation/incidence/prevalence/ magnitude of ageism	Quantitative - correlational	Cross-sectional	Clustered	103085	18-64	52%	People with health-related limitations	18-44 vs. 45-64
(Haefner, 1977a)	United States of America	Manifestation/incidence/prevalence/ magnitude of ageism	Quantitative - experimental	Cross-sectional	Simple Random	286	M=44	11%	Employers	25 year old worker vs 55 year old worker
(Haefner, 1977b)	United States of America	Manifestation/incidence/prevalence/ magnitude of ageism	Quantitative - correlational	Cross-sectional	Clustered	588	M=42.1	36%	Employees	25 years old or 55 years old
(Hall & Deahl, 1995)	United Kingdom	Manifestation/incidence/prevalence/ magnitude of ageism	Quantitative - descriptive	Longitudinal	Entire population	227	16-79(M=32, SD=12)	42%	All the available case notes of new patients seen in an Emergency Clinic in the first 165 days of 1991	16-29
(Harwood, Giles, Clement, Pierson, & Fox, 1994)	China, United States of America	Manifestation/incidence/prevalence/ magnitude of ageism	Quantitative - correlational	Cross-sectional	Convenience	191 (China sample: 93; US sample: 98)	China: M=23.42, SD=3.86; US: M=23.37, SD=1.80	China sample: 40.8%; US sample: 89.7&	University students	young, middle-aged and older targets

Author(s), year	Country	Main purpose of the study	Type of Research	Study design timeframe	Sampling approach	Sample size	Age [Range (Mean, SD)]	Sex (% female)	Population	Target age group studied
(Harwood et al., 2001)	Australia, China, Philippines, Thailand	Manifestation/incidence/prevalence/ magnitude of ageism	Quantitative - experimental	Cross-sectional	Convenience	615	over the age of 48 (M=68.79. SD=6.71)	Ranged from 66.7% (Australia) to 44.7% (China)	Adults	young (20–30 years old), middle-aged (45–55 years old) and older (65–85 years old) adults
(Harwood et al., 1996)	Australia, China, New Zealand, Philippines, United States of America, Republic of Korea	Manifestation/incidence/prevalence/ magnitude of ageism	Quantitative - correlational	Cross-sectional	Convenience	1073	Not reported	65.4% in the US 50.5% in China	College students	Young:20-30 year olds, middle-aged: 45-55, old: 65-85 year olds
(Harwood, Giles, Fox, Ryan, & Williams, 1993)	United States of America	Manifestation/incidence/prevalence/ magnitude of ageism	Quantitative - experimental	Cross-sectional	Convenience	222	M=19.06	62%	Students	28 years-old (vs. 69)
(Hatta, Higashikawa, & Hatta, 2010)	Japan	Manifestation/incidence/prevalence/ magnitude of ageism	Quantitative - descriptive	Cross-sectional	Convenience	1336	Reported separately for men and women for the following age groups 20-30, 41-50, 51-60, 61-70, 71-80, 81-90	20-30: 54.3%; 41-50: 66.9%; 51-60: 62%; 61-70: 57.6%; 71-80: 34.5%; 81-90: 47.7%	College students adults over 40 years old	Age groups of participants
(Hayes & Phill Johnson, 2018)	United States of America	Manifestation/incidence/prevalence/ magnitude of ageism	Quantitative - descriptive	Cross-sectional	Convenience	1080	22+ years old	Not reported	Professional academic librarians	Millennial generation (22–36 years old); Generation X (37–52 years old): Baby Boomer (53–71 years old)
(He, Ebner, & Johnson, 2011)	United States of America	Manifestation/incidence/prevalence/ magnitude of ageism, Determinants of ageism	Quantitative - experimental	Cross-sectional	Convenience	49	Younger adults: 19-29 (M=22.2, SD = 2.9), Older adults: 63–92 (M=73.9, SD = 7.8)	Younger adults: 60% Older adults: 71%	University students and community dwelling older adults	Young faces, 18-31 years
(Hebl, Ruggs, Singletary, & Beal, 2008)	United States of America	Manifestation/incidence/prevalence/ magnitude of ageism, Determinants of ageism	Quantitative - experimental	Cross-sectional	Convenience	208	18 -77 (M=39.41, SD=17.44)	51%	Adults recruited from various places (e.g., university campus, malls, coffee shops, book stores).	20 and 40 and 60-year-old faces on slender, large, and very large-sized bodies.

Author(s), year	Country	Main purpose of the study	Type of Research	Study design timeframe	Sampling approach	Sample size	Age [Range (Mean, SD)]	Sex (% female)	Population	Target age group studied
(Hehman & Bugental, 2013)	United States of America	Consequences of ageism	Quantitative - experimental	Cross-sectional	Convenience	81	17-22(M=18.72, SD=1.06)	49%	University students	17-22
(Hendrick, Knox, Gekoski, & Dyne, 1988)	Canada	Manifestation/incidence/prevalence/ magnitude of ageism	Quantitative - experimental	Cross-sectional	Convenience	80	18-22 (M=19.2)	100%	University students	A 21-year old or an average non-institutionalized 71-year old
(Hendrick et al., 1988)	Canada	Manifestation/incidence/prevalence/ magnitude of ageism	Quantitative - experimental	Cross-sectional	Convenience	120	17-24 (M=19.1)	50%	University students	A 21-year old or an average non-institutionalized 71-year old
(Heyman & Gutheil, 2008)	United States of America	Interventions to tackle ageism	Qualitative - not specified	Cross-sectional	Convenience	10	25-62 (M=43.5)	90%	Staff working at a center that operates a shared site intergenerational program	children, older adults
(Heyman & Gutheil, 2008)	United States of America	Interventions to tackle ageism	Qualitative - not specified	Cross-sectional	Convenience	6	75-95 (M=84.2)	83.30%	Older adults attending a center that operates a shared site intergenerational program	children, older adults
(Heyman & Gutheil, 2008)	United States of America	Interventions to tackle ageism	Qualitative - not specified	Cross-sectional	Convenience	10	35-70 (M=46.5)	70%	Caregivers/parents of older adults/children attending a center that operates a shared site intergenerational program	children, older adults
(Heyman & Gutheil, 2008)	United States of America	Interventions to tackle ageism	Qualitative - not specified	Cross-sectional	Convenience	10	8-12 (M=10.4)	70%	children attending a center that operates a shared site intergenerational program	children, older adults

Author(s), year	Country	Main purpose of the study	Type of Research	Study design timeframe	Sampling approach	Sample size	Age [Range (Mean, SD)]	Sex (% female)	Population	Target age group studied
(Hnilica, 2011)	Multiple European countries (more than 20)	Consequences of ageism	Quantitative - correlational	Cross-sectional	Stratified	39,552 in the year 2002, 46,331 in the year 2004, and 37,934 in the year 2006 (exact distribution based on age is not reported)	>15	Not reported	Adults	Own age
(Hui et al., 2014)	China	Manifestation/incidence/prevalence/ magnitude of ageism	Quantitative - descriptive	Cross-sectional	Convenience	594 from four age groups: 149 emerging adults; 148 young adults; 148 middle-aged working adults; and 149 older adults	18-25 (emerging adults); 26-40 (young adults); 41-60 (middle-aged working adults); more than 61 (older adults)	43.3%(emerging adults); 66.7%(young adults); 57.3% (middle-aged working adults); 70.7% (older adults)	University students , people working in arts industries, living in day centres for seniors	Ages between 20 and 90
(Hummert, Garstka, & Shaner, 1995)	United States of America	Manifestation/incidence/prevalence/ magnitude of ageism	Quantitative - descriptive	Cross-sectional	Convenience	40 younger adults	Younger adults: M=20.5, 19-23	50%	University students	Young (19-23), middle aged (31-53), older adults (60-90)
(Hummert, 1990)	United States of America	Manifestation/incidence/prevalence/ magnitude of ageism	Quantitative - descriptive	Cross-sectional	Convenience	37 (+37 who focused on older adults)	Not reported	Not reported	College students	young people vs. older adults
(Hummert, 1990)	United States of America	Manifestation/incidence/prevalence/ magnitude of ageism	Quantitative - descriptive	Cross-sectional	Convenience	37 (+44 responded about older adults)	Not reported	Not reported	College students	young people vs. older adults
(Hung, Giles, & Moody, 1991)	New Zealand	Manifestation/incidence/prevalence/ magnitude of ageism, Determinants of ageism	Quantitative - experimental	Cross-sectional	Convenience	437	18-23 (M=19.2, SD=1.2)	64.30%	University students	16, 21, 26, 31, 41, ,51, 61, 71, 81, and 91 years
(Inbar, Doron, & Ohry, 2012)	Israel	Determinants of ageism, Manifestation/incidence/prevalence/ magnitude of ageism	Quantitative - experimental	Cross-sectional	Convenience	102	Not reported	84%	Physiotherapists	32 VS. 81
(Ivey, Wieling, & Harris, 2000)	United States of America	Manifestation/incidence/prevalence/ magnitude of ageism, Determinants of ageism	Quantitative - experimental	Cross-sectional	Convenience, Simple Random	359 including 128 non-therapists, 113 therapists in training, and 118 therapists	Non-therapists (M=22.5, SD=3.3); Therapists in training (M=37.75, SD=8.47); Therapists (M=48.17, SD=12.24)	Non-therapists: 66%; Therapists in training: 78%; Therapists: 52%	University students and therapists in training	older married couple (Male age 74, female age 69) and younger married couple (female age 29, male age 34)

**Author(s), year**	**Country**	**Main purpose of the study**	**Type of Research**	**Study design timeframe**	**Sampling approach**	**Sample size**	**Age [Range (Mean, SD)]**	**Sex (% female)**	**Population**	**Target age group studied**
(Jackson & Bennion, 2019)	United States of America	Manifestation/incidence/prevalence/ magnitude of ageism	Quantitative - experimental	Cross-sectional	Convenience	80	18-28 (M = 19.89, SD = 1.68)	65%	University students	Children vs. adults
(James & Haley, 1995)	United States of America	Manifestation/incidence/prevalence/ magnitude of ageism	Quantitative - experimental	Cross-sectional	Simple Random	371	M=50.86, SD=9.65	26%	Doctoral-level psychologists	35 vs. 70
(Jarrott & Gigliotti, 2011)	United States of America	Interventions to tackle ageism	Quantitative - experimental	Longitudinal	Convenience	40	19-52	97.50%	Formal network members from the adults' and children's programs	children, older adults
(Jarrott & Gigliotti, 2011)	United States of America	Interventions to tackle ageism	Qualitative - not specified	Longitudinal	Convenience	21	19-53	95.20%	Formal network members from the adults' and children's programs	children, older adults
(Johnston & Alozie, 2001)	United States of America	Manifestation/incidence/prevalence/ magnitude of ageism	Quantitative - descriptive	Cross-sectional	Entire population	prefiles of 5,715 drug offenders	16-64 (M=28)	Not reported	Drug offenders	different ages between 16 to 64
(Joshi, 2013)	Armenia, Georgia, Indonesia, Kyrgyzstan, Nepal, Sri Lanka, Azerbaijan, Bangladesh, India, Japan, Republic of Korea, Thailand, Mongolia, Singapore	Interventions to tackle ageism	Quantitative - descriptive	Cross-sectional	Purposive or judgement	14 countries-relevant entire population in each	18-60+	Not reported	Members of parliament from 14 countries	<30, <35, <50
(Kainer, 2016)	Canada	Manifestation/incidence/prevalence/ magnitude of ageism	Qualitative - case study	Cross-sectional	Snowball	14	22-36	100%	Young women organizers working in unions and labour federations	22-36
(Kane, 2004)	United States of America	Manifestation/incidence/prevalence/ magnitude of ageism	Quantitative - correlational	Cross-sectional	Convenience	173	Not reported	79.70%	BSW and MSW students	38 years vs 72 years
(Kane, Green, & Jacobs, 2010)	United States of America	Manifestation/incidence/prevalence/ magnitude of ageism	Quantitative - experimental	Cross-sectional	Convenience	207	19-58 (M=30.77)	87.40%	University students	Younger and older
(Kane, Jacobs, & Sherman, 2015)	United States of America	Manifestation/incidence/prevalence/ magnitude of ageism	Quantitative - experimental	Cross-sectional	Convenience	324	19-61 (M=26.59, SD=8.53)	69%	University students	28 yrs old vs 68 year old

Author(s), year	Country	Main purpose of the study	Type of Research	Study design timeframe	Sampling approach	Sample size	Age [Range (Mean, SD)]	Sex (% female)	Population	Target age group studied
(Kane, Jacobs, & Hawkins, 2013)	United States of America	Manifestation/incidence/prevalence/ magnitude of ageism	Quantitative - experimental	Cross-sectional	Convenience	375	18-60 (M=26.53)	70%	University students	28-year-old vs. 72-year-old
(Karbon & Martin, 1992)	United States of America	Manifestation/incidence/prevalence/ magnitude of ageism, Determinants of ageism	Quantitative - experimental	Cross-sectional	Convenience	67	46-74 months old (M=60.7month, SD=6.95)	52%	English speaking pre-school children	pre-schoolers vs adults
(Kastenbaum & Arrt, 1972)	United States of America	Manifestation/incidence/prevalence/ magnitude of ageism	Qualitative - not specified	Cross-sectional	Convenience	43 (gerontology specialists); 31 (general, non-gerontologic sample)	20-60 (gerontology specialists); 20-65 (general, non-gerontologic sample)	62.7% (gerontology specialists); 32.2% (general, non-gerontologic sample)	University students in gerontology and employees of a state agency (general, non-gerontologic sample).	all ages from 20-65
(Kelan, 2014)	United Kingdom	Manifestation/incidence/prevalence/ magnitude of ageism	Qualitative - not specified	Cross-sectional	Purposive or judgement	32	Not reported (young professionals born between 1977 and 1985)	50%	Employees	young professionals, older professionals
(Kellner & Waterhouse, 2011)	Australia	Manifestation/incidence/prevalence/ magnitude of ageism	Qualitative - content analysis	Cross-sectional	Convenience	1259	15-24	64%	Workplace dismissals reported to YWAS over a three year period from 2002 to 2005	15-24
(Kidwell & Booth, 1977)	United States of America	Consequences of ageism, Manifestation/incidence/prevalence/ magnitude of ageism, Measurement of ageism	Quantitative - correlational	Cross-sectional	Quota	440	19+	Not reported	Members of church groups	19-24, 25-34, 35-44, 45-54, 55-64, 65-74, 75 and older
(Kite, Stockdale, Whitley, & Johnson, 2005)	Not reported	Manifestation/incidence/prevalence/ magnitude of ageism, Determinants of ageism	Quantitative - descriptive	Cross-sectional	Entire population	232 effect sizes	21-35 young, 36-54 middle aged, >55-old	Not clear	Relevant articles that met explicit criteria	young (21-35 years old), middle age (36-54 years old), old (55 years or older)
(Kite & Johnson, 1988)	Not reported	Manifestation/incidence/prevalence/ magnitude of ageism	Quantitative - descriptive	Cross-sectional	Not applicable	43 effect sizes	Not reported	Not relevant	Not reported	Young people, older people
(Kmicinska, Zaniboni, Truxillo, Fraccaroli, & Wang, 2016)	Italy	Determinants of ageism	Quantitative - correlational	Longitudinal	Convenience	114	18–66 (M=37.50, SD=11.11)	46.70%	Employees and managers.	24-34, 50-60 year olds
(Knox, Gekoski, & Kelly, 1995)	United States of America	Measurement of ageism	Quantitative - correlational	Cross-sectional	Convenience	600	17-23	50%	University students	mid-twenties, mid-forties

Author(s), year	Country	Main purpose of the study	Type of Research	Study design timeframe	Sampling approach	Sample size	Age [Range (Mean, SD)]	Sex (% female)	Population	Target age group studied
(Knox et al., 1995)	United States of America	Measurement of ageism	Quantitative - correlational	Cross-sectional	Convenience	800	17-23	50%	University students	mid-twenties, mid-forties
(Kogan, 1979)	United States of America	Manifestation/incidence/prevalence/ magnitude of ageism, Determinants of ageism	Quantitative - experimental	Cross-sectional	Convenience	150	18-21, 22-28, 29-38, 39-55, and 56-76	50%	College students and older adults from a senior citizens center	adolescent, young, middle-aged, older adult, aged adult
(Kohfeldt & Langhout, 2011)	United States of America	Interventions to tackle ageism	Qualitative - ethnography	Cross-sectional	Convenience	17	fifth graders	65%	Fifth grade students	fifth graders
(Kornadt, Hess, Voss, & Rothermund, 2016)	Germany	Manifestation/incidence/prevalence/ magnitude of ageism, Determinants of ageism	Quantitative - correlational	Longitudinal	Simple Random	222	30–49 (M=39.79, SD=5.50)	47%	People living in two middle-sized cities in Germany	The age of the individual participant with individual participants aged 30-49
(Kornadt, Meissner, & Rothermund, 2016)	Germany	Manifestation/incidence/prevalence/ magnitude of ageism	Quantitative - experimental	Cross-sectional	Convenience	31 younger, 26 middle aged, and 33 older adults	19–29 (M = 22.65, SD = 2.81); 32–59 (M = 49.11, SD = 7.88); 60–88 (M = 67.58, SD = 6.20)	59%	University students and older and middle-aged adults	younger and older persons
(Kuhlmann, Kornadt, Bayen, Meuser, & Wulff, 2017)	Germany	Manifestation/incidence/prevalence/ magnitude of ageism	Quantitative - experimental	Cross-sectional	Convenience	69 younger participants; 74 older participants	Younger participants: 18-26 (M=22.03). Older participants: 60-84 (M=70.17)	Younger participants: 66.2%. Older participants: 69.6%	University students, community dwellers	No specific target age; only "young adult" and "old adult" specified. Participants also indicated the age (in years) they had thought of while rating (younger participants: M = 22.15 [17–30], SD = 2.14, for “young adult” and 67.58 [45–80], SD = 9.09, for “old adult;” older participants: M = 24.15 [12–35], SD = 4.21, and 70.70

Author(s), year	Country	Main purpose of the study	Type of Research	Study design timeframe	Sampling approach	Sample size	Age [Range (Mean, SD)]	Sex (% female)	Population	Target age group studied
										[50–100], SD = .01, respectively),
(Kuhlmann, Bayen, Meuser, & Kornadt, 2016)	Germany	Manifestation/incidence/prevalence/ magnitude of ageism	Quantitative - experimental	Cross-sectional	Convenience	144	18–26 (M= 21.6, SD=2.1) 60–84 (M=70.1, SD=5.2)	Younger adults: 64.6% Older adults: 70.8%	University students and community-dwelling older adults	23 yrs
(Kuhlmann et al., 2016)	Germany	Manifestation/incidence/prevalence/ magnitude of ageism	Quantitative - experimental	Cross-sectional	Convenience	48	17–25 (M=20.88, SD 2.02)	68.80%	University students	23 yrs
(Lahad & Madsen, 2016)	Denmark	Manifestation/incidence/prevalence/ magnitude of ageism	Qualitative - content analysis	Cross-sectional	Purposive or judgement	Not reported	40+	100%	40+ mothers	40+ mothers
(Larme, 1997)	Peru	Manifestation/incidence/prevalence/ magnitude of ageism	Qualitative - ethnography	Cross-sectional	Purposive or judgement	11 families, including 23 children- I am not sure	Not clear	Not reported	Families with children below the age of 7	Children less than one year old; children between 1 and 3 years old; children 4 to 6 years old
(Larme, 1997)	Peru	Manifestation/incidence/prevalence/ magnitude of ageism	Quantitative - descriptive	Cross-sectional	Purposive or judgement	178 sympton reports analysed from 23 children	Below 7 years of age	Not reported	Children under 7	Children less than one year old; children between 1 and 3 years old; children 4 to 6 years old

Author(s), year	Country	Main purpose of the study	Type of Research	Study design timeframe	Sampling approach	Sample size	Age [Range (Mean, SD)]	Sex (% female)	Population	Target age group studied
(Larson & Diaz, 2012)	United States of America	Manifestation/incidence/prevalence/ magnitude of ageism	Quantitative - correlational	Cross-sectional	Convenience	Not reported	Not reported	Not reported	Faculty recruitments and departure data available at MIT	Younger faculty (prospective hires)
(LaVeist, Rolley, & Diala, 2003)	United States of America	Manifestation/incidence/prevalence/ magnitude of ageism	Quantitative - descriptive	Cross-sectional	Simple Random	3080	18-30, 31-50, 51-65, 65+	Not reported	Adults aged 18+	18-30; 31-50, 51-65, 65+
(Lee & Pillutla, 2015)	Singapore	Manifestation/incidence/prevalence/ magnitude of ageism, Determinants of ageism	Quantitative - experimental	Cross-sectional	Convenience	121	(M=30.77, SD=10.01)	0%	Adult males with work experience	25 vs. 50
(Levin, 1988)	United States of America	Determinants of ageism, Manifestation/incidence/prevalence/ magnitude of ageism	Quantitative - experimental	Cross-sectional	Convenience	510	SFSU: M=20.83<br>ETSU: M=21.16<br>SSC: M=21.28	Not reported	University students	25 years, 52, 73
(Lin, Ankudowich, & Ebner, 2017)	United States of America	Manifestation/incidence/prevalence/ magnitude of ageism, Determinants of ageism	Quantitative - experimental	Cross-sectional	Convenience	27 younger participants; 23 older participants	Younger participants: 18-27 (M=21.07, SD=2.40).<br>Older participants: 61-86 (M=72.91, SD=7.31)	Younger participants: 62.9%<br>Older participants: 47.8%	University students and community dwelling older adults	average young ("between the ages of 18 and 30 years") and the average old ("over the age of 60 years) target person; as well as the participant itself
(Lin et al., 2017)	United States of America	Manifestation/incidence/prevalence/ magnitude of ageism, Determinants of ageism	Quantitative - experimental	Cross-sectional	Convenience	50 younger participants; 51 older participants	Younger participants: 18-22 (M=18.81, SD=0.98).<br>Older participants: 60-92 (M=73.76, SD=7.70)	Younger participants: 50%;<br>Older participants: 52%.	University students and community dwelling older adults	average young ("between the ages of 18 and 30 years") and the average old ("over the age of 60 years) target person; as well as the participant itself
(Lindner & Nosek, 2014)	United States of America	Interventions to tackle ageism	Quantitative - experimental	Cross-sectional	Convenience	1590	18-80 (M=30.2, SD=12.7)	65%	Adults	31 vs. 54
(Linville, 1982)	United States of America	Manifestation/incidence/prevalence/ magnitude of ageism	Quantitative - experimental	Cross-sectional	Convenience	22	Not reported	0%	University students	college-aged males ; males in their 60s and 70s.

Author(s), year	Country	Main purpose of the study	Type of Research	Study design timeframe	Sampling approach	Sample size	Age [Range (Mean, SD)]	Sex (% female)	Population	Target age group studied
(Linville, 1982)	United States of America	Manifestation/incidence/prevalence/ magnitude of ageism	Quantitative - experimental	Longitudinal	Convenience	33	Not reported	0%	University students	college-aged males ; males in their 60s and 70s.
(Loretto & Duncan, 2000)	United Kingdom	Manifestation/incidence/prevalence/ magnitude of ageism	Quantitative - correlational	Cross-sectional	Convenience	460	17-29	45%	University students	17-29; younger employees
(Lucas & Keegan, 2008)	United States of America	Manifestation/incidence/prevalence/ magnitude of ageism	Qualitative - not specified	Cross-sectional	Convenience	15	Not reported	Not reported	Managers	Young workers (16-17, 18-21, 22+)
(Luoh & Tsaur, 2014)	China	Determinants of ageism, Manifestation/incidence/prevalence/ magnitude of ageism	Quantitative - experimental	Cross-sectional	Convenience	447	193 middle aged: 40-59, M=48.3; 254 young 18-39, M=29.4	Not reported	University students and community dwelling adults who travelled abroad	Young-approx. 25; Middle-aged approx-45
(Luoh & Tsaur, 2011)	China	Manifestation/incidence/prevalence/ magnitude of ageism, Determinants of ageism	Quantitative - experimental	Cross-sectional	Convenience	406 (277 young participants; 129 middle-aged participants)	Young participants: 19-39 (M=28.1). Middle-aged participants: 40-59 (M=46.8).	Not reported	University students and community dwelling adults who travelled abroad	"middle-aged" (apprx. 40 years old) and "young" (apprx. 20 years old) server as per images
(Luszcz, 1986)	Australia	Manifestation/incidence/prevalence/ magnitude of ageism, Determinants of ageism	Quantitative - experimental	Cross-sectional	Convenience	180 (60 adolescents, 60 middle-aged adults, 60 older adults)	Adolescents (M=17.6, SD=0.5); Middle aged adults (M=46.2, SD=5.3); Older adults (M=69.8, SD=6.4)	Adolescents: 70%; Middle-aged adults: 55%; Older adults: 51.6%	Community dwelling adults	adolescents (age specified as 15 to 20 years), middle-aged adults (age specified as 40 to 55 years), or elderly adults (age specified as 65 years or more).
(Luszcz & Fitzgerald, 1986)	Australia	Manifestation/incidence/prevalence/ magnitude of ageism	Quantitative - correlational	Cross-sectional	Convenience	90 including 30 per age group (adolescents, middle aged and older adults)	Adolescents: (M=16 SD=0.5), Middle aged: (M=46, SD=6.2), Older adults: (M=68, SD=6.6)	Adolescents-53%, Middle-aged-63%; Older - 53%	Adolescents and community dwelling middle-aged and older adults	Self+ Adolescents: 15-19, Middle-aged: 40-55, Older adults: >60. An additonal age breakdown was used: those aged 15 to 19, 20 to 29, 30 to 39,40 to 49,

Author(s), year	Country	Main purpose of the study	Type of Research	Study design timeframe	Sampling approach	Sample size	Age [Range (Mean, SD)]	Sex (% female)	Population	Target age group studied
										50 to 59, and over 60 years.
(Lyons & Schweitzer, 2017)	Canada	Manifestation/incidence/prevalence/ magnitude of ageism	Qualitative - not specified	Cross-sectional	Convenience	105	M=39	54%	Employees	Baby Boomer (aged 47–65 at the time of the study), Young generations - Generation Xers (aged 32–46), and Millennials (aged 31 and younger)
(Mangan & Johnston, 1999)	Australia	Manifestation/incidence/prevalence/ magnitude of ageism	Quantitative - not specified	Longitudinal	Entire population	Not reported	Not reported	Not reported	Youth	Youth (15-19 yrs)
(Marchiondo, Gonzales, & Ran, 2016)	United States of America	Measurement of ageism, Manifestation/incidence/prevalence/ magnitude of ageism	Qualitative - not specified	Cross-sectional	Convenience	106	18–39 (M=28.9, SD = 5.4)	51%	U.S. workers	Young employees
(Marchiondo et al., 2016)	United States of America	Measurement of ageism	Quantitative - correlational	Cross-sectional	Convenience	294	18–30 (M=25.2, SD = 2.7)	40%	U.S. workers	Young employees
(Marchiondo et al., 2016)	United States of America	Measurement of ageism	Quantitative - correlational	Cross-sectional	Convenience	403	18–30 (M=25.2, SD = 2.9)	39%	U.S. workers	Young employees
(Marchiondo et al., 2016)	United States of America	Measurement of ageism	Quantitative - correlational	Cross-sectional	Convenience	407	31–49	46%	Workers	Middle aged employees
(Marchiondo et al., 2016)	United States of America	Manifestation/incidence/prevalence/ magnitude of ageism	Quantitative - correlational	Cross-sectional	Convenience	Young: 403; middle aged: 407	Young: 18–30 (M=25.2, SD = 2.9); middle aged: 31–49	Young: 39%; middle aged: 46%	U.S. workers	Young and middle aged employees
(Marcus & Fritzsche, 2014)	United States of America	Manifestation/incidence/prevalence/ magnitude of ageism, Determinants of ageism	Quantitative - experimental	Cross-sectional	Convenience	724	(M=18.73;SD=1.64)	64%	University students	Mid-twenties

Author(s), year	Country	Main purpose of the study	Type of Research	Study design timeframe	Sampling approach	Sample size	Age [Range (Mean, SD)]	Sex (% female)	Population	Target age group studied
(Matheson & Kuehne, 2000)	Canada	Manifestation/incidence/prevalence/ magnitude of ageism	Quantitative - correlational	Cross-sectional	Simple Random	68	65-85 (M=75.3 for males; M=72 for females)	54.40%	Young people	young people, specified as those in their late teens and twenties.
(Matyi & Drevenstedt, 1989)	United States of America	Manifestation/incidence/prevalence/ magnitude of ageism	Quantitative - experimental	Cross-sectional	Convenience	375	Not reported	49%	University students	25 vs 74 years old
(McCann, Dailey, Giles, & Ota, 2005)	United States of America	Manifestation/incidence/prevalence/ magnitude of ageism	Quantitative - experimental	Cross-sectional	Convenience	137	16-28 (M=19.97, SD=1.57)	50%	University students	young adults, middle-aged adults, and older adults, with respondents having the possibility to define the target age range of the specified targets
(McCann & Giles, 2006)	Thailand, United States of America	Theory development, Manifestation/incidence/prevalence/ magnitude of ageism	Quantitative - descriptive	Cross-sectional	Convenience	348 (168 from the US)	US: M=23.15, SD=3.79, Thailand: M=29.22, SD=2.84	67%	Nonmanagerial bankers	18-34 vs. 50+
(McCann & Keaton, 2013)	United States of America, Thailand	Manifestation/incidence/prevalence/ magnitude of ageism	Quantitative - correlational	Cross-sectional	Convenience	267	18-33 (M = 22.49, SD = 2.71)	US: 80%; Thailand: 72%	University students	Young workers
(McClellan & Beggan, 2017)	United States of America	Manifestation/incidence/prevalence/ magnitude of ageism	Qualitative - not specified	Cross-sectional	Purposive or judgement	26	27 - 64 (M=42.19, SD = 11.82)	85%	Librarians	Young librarians, older librarians
(McNamara, Pitt-Catsouphes, Sarkisian, Besen, & Kidahashi, 2016)	United States of America	Determinants of ageism	Quantitative - correlational	Cross-sectional	Convenience	544	31-59	64.20%	Employees	"Relatively younger" employees (workers who were at least ten years younger) and "approximately same age" employees, "relatively older" employees
(Meinich & Sang, 2018)	Norway	Manifestation/incidence/prevalence/ magnitude of ageism	Qualitative - not specified	Cross-sectional	Purposive or judgement	20	Not reported (reported by generations: 35% baby boomer, 30% generation X, 35% millennial)	10%	Employees	Millennial, Generation X, Baby Boomer

Author(s), year	Country	Main purpose of the study	Type of Research	Study design timeframe	Sampling approach	Sample size	Age [Range (Mean, SD)]	Sex (% female)	Population	Target age group studied
(Menec & Perry, 1995)	Canada	Manifestation/incidence/prevalence/ magnitude of ageism	Quantitative - experimental	Cross-sectional	Convenience	249	19-29	62.65%	University students	25-35 years, 55-65 years
(Meshel & McGlynn, 2004)	United States of America	Interventions to tackle ageism	Quantitative - experimental	Longitudinal	Convenience	17 older adults, 63 younger participants	Older adults=60-75+; Younger= 11-13	71%-older adults, 54%-younger participants	Older adults from a senior citizen center and middle school students	middle school adolescents (age 11–13) (and older adults 60+)
(Miller, Kaspin, & Schuster, 1990)	United States of America	Manifestation/incidence/prevalence/ magnitude of ageism	Qualitative - content analysis	Cross-sectional	Purposive or judgement	53 federal court ADEA cases	Not reported	Not reported	federal court ADEA cases in which performance appraisal evidence was central to the case outcome.	under 50 yrs, over 50 yrs
(Mooney, 2016)	New Zealand	Manifestation/incidence/prevalence/ magnitude of ageism	Qualitative - not specified	Cross-sectional	Convenience	31	21-70	52%	Ordinary people in hospitality	youth
(Moore, 2018)	Not reported	Manifestation/incidence/prevalence/ magnitude of ageism	Qualitative - content analysis	Cross-sectional	Purposive or judgement	4 books	Not reported	Not reported	Two groups of adult characters parents and school professionals in four YA sexual assault narratives	Young women
(Morgeson & Bull, 2008)	Not reported	Manifestation/incidence/prevalence/ magnitude of ageism	Qualitative - not specified	Cross-sectional	Not applicable	21 articles	Reported for each individual study	Not reported	Reported for each individual study with 5 studies conducted in the field and the rest in the lab	Reported for each individual study (overall 18 and over)
(Netz & Ben-Sira, 1993)	Israel	Manifestation/incidence/prevalence/ magnitude of ageism	Quantitative - descriptive	Cross-sectional	Convenience	62 families including 3 age groups=186	Young: M=18.92, SD=4.92; Middle-age: M=44.98, SD=8.92; Older adults: M=68.8, SD=8.04	Young=60%; Middle-age=70%; Older adults=81%. Not fully reported for all participants	Three-generation families	Ideal person, youth, adult, old person
(Ng, Gilles, & Moody, 1991)	New Zealand	Manifestation/incidence/prevalence/ magnitude of ageism	Quantitative - experimental	Cross-sectional	Convenience	437	18-23 (M=19.2, SD=1.2)	64%	University students	16, 21, 26, 31, , 41, 51 , 61, 71, 81, 91

**Author(s), year**	**Country**	**Main purpose of the study**	**Type of Research**	**Study design timeframe**	**Sampling approach**	**Sample size**	**Age [Range (Mean, SD)]**	**Sex (% female)**	**Population**	**Target age group studied**
(Ng, Liu, Weatherall, & Loong, 1997)	New Zealand	Manifestation/incidence/prevalence/ magnitude of ageism	Quantitative - experimental	Cross-sectional	Convenience	100 (50 european, 50 chinese).	14-46	51%	Community dwelling adults of either European or Chinese descent.	own age peers (14-46), older family and non-family members (65-85)
(Ng & Chan, 1996)	China	Manifestation/incidence/prevalence/ magnitude of ageism	Qualitative - not specified	Cross-sectional	Convenience	297	15-16	50%	High school students	Children-10 years, youth-20years, middle-aged-40 years, elderly-70 years
(Noels & Turay, 1999)	United States of America	Manifestation/incidence/prevalence/ magnitude of ageism, Consequences of ageism	Quantitative - correlational	Cross-sectional	Convenience	young: 65; older: 68	young: 17-28 (M=20.52, SD = 1.75); older: 60+ (M=67.60, SD = 4.41)	young: 69%; older: 53%	Not reported	younger people (17 to 35 years); older people (65>=)
(Noels & Turay, 1999)	United States of America, China	Manifestation/incidence/prevalence/ magnitude of ageism, Consequences of ageism	Quantitative - correlational	Cross-sectional	Convenience	China: 68; USA: 68 (same sample as study 1)	China: 60-80 (M=66.82, SD = 4.29); USA: 60-80 (M=67.60, SD = 4.41)	China: 43%; USA: 53%	Not reported	younger people (17 to 35 years), older people 65+
(Öberg & Tornstam, 2001)	Sweden	Manifestation/incidence/prevalence/ magnitude of ageism	Quantitative - descriptive	Cross-sectional	Simple Random	1250	20-85	55%	A random sample of men and women	All ages
(O'Connell & Rotter, 1979)	United States of America	Manifestation/incidence/prevalence/ magnitude of ageism	Quantitative - experimental	Cross-sectional	Convenience	306	Not reported	49.67%	College students	25, 50, and 75 year olds
(Ota, Giles, & Gallois, 2002)	Australia, Japan	Manifestation/incidence/prevalence/ magnitude of ageism	Quantitative - experimental	Cross-sectional	Convenience	155 (Japanese); 171 (Australian)	Japan: 19-26 (M=20.53, SD=0.98); Australia: 17-27 (M=18.94, SD=2.26)	Japan: 54.8%; Australia: 54.3%	University students	younger, middle-aged, and older adults as defined by participants
(Ota, McCann, & Honeycutt, 2012)	Thailand, Japan	Manifestation/incidence/prevalence/ magnitude of ageism	Quantitative - descriptive	Cross-sectional	Convenience	244 (of these 131 Thai)	Japanese (M=19.18, SD=1.05) Thai (M=21.05, SD=1.04)	63.7% Japanese, 76.3% Thai	University students	Three age groups: young, middle age, old

Author(s), year	Country	Main purpose of the study	Type of Research	Study design timeframe	Sampling approach	Sample size	Age [Range (Mean, SD)]	Sex (% female)	Population	Target age group studied
(Ota, Giles, & Somera, 2007)	United States of America, Japan, Philippines	Manifestation/incidence/prevalence/ magnitude of ageism	Quantitative - correlational	Cross-sectional	Convenience	509 participants (USA: 67 younger and 87 older; Japan: 102 young and 102 older; Philippines: 51 young and 100 older)	USA: (younger adults M= 20.51, SD = 1.74; older adults M=69.3; SD = 5.63); Japan: (younger adults M=18.64, SD = 1.45; older adults M= 69.41; SD = 5.40); Philippines: (younger adults M=18.9, SD = 1.45; older adults M=71.97, SD = 6.79)	USA: 67% young female and 43% older female; Japan: 37% young female and 48% older female; Philippines: 76% young female and 65% older females.	University students and community dwelling older adults	younger adults (18-28 years old) and older adults aged 65 and older
(Oudshoorn, Neven, & Stienstra, 2016)	Netherlands	Manifestation/incidence/prevalence/ magnitude of ageism	Qualitative - case study	Cross-sectional	Purposive or judgement	5 + documents (internal progress reports, the business plan, conference papers, master theses of students who did their graduation projects on KidCom, the manual, and the collages developed during the design process).	Not reported	40%	Project leader, product and industrial manager, and members of the project team involved in the research and design of KidCom	Young girls
(Pak & Bass, 2014)	United States of America	Manifestation/incidence/prevalence/ magnitude of ageism	Quantitative - experimental	Cross-sectional	Convenience	107 (60 younger adults; 47 older adults)	younger adults: M=18.6, SD=0.9; older adults: M=72.7, SD=5.3	younger adults: 61.6%; older adults: 53.2%	University students and community-dwelling older adults.	young female, young male, older female, older male
(Palmeira & Musso, 2017)	Brazil, Italy	Manifestation/incidence/prevalence/ magnitude of ageism	Quantitative - descriptive	Cross-sectional	Convenience	Brazil: 89; Italy: 112	up to 30 years old, older than 30 years old	Brazil: 48%; Italy: 78%-82%	Vacationers of seaside retailing services and University students	younger people, older people
(Palumbo, Adams, Hess, Kleck, & Zebrowitz, 2017)	Italy	Manifestation/incidence/prevalence/ magnitude of ageism	Quantitative - correlational	Cross-sectional	Purposive or judgement	240 generalization faces	younger faces: 18-31(M=23.06, SD=3.22), older faces: 65-91 (M=73.42, SD=5.41)	50%	Generalization faces included older and younger neutral expression faces	Young 18-31 vs. Old 65-91

Author(s), year	Country	Main purpose of the study	Type of Research	Study design timeframe	Sampling approach	Sample size	Age [Range (Mean, SD)]	Sex (% female)	Population	Target age group studied
(Palumbo et al., 2017)	Italy	Manifestation/incidence/prevalence/ magnitude of ageism	Quantitative - correlational	Cross-sectional	Convenience	200	100 younger adults, 100 older adults	50%	University students and community dwelling older adults	Young 18-31 vs. Old 65-91
(Perry, 1994)	United States of America	Determinants of ageism	Quantitative - experimental	Cross-sectional	Convenience	20	Not reported	25%	University students	20-25 vs. 45-50
(Perry et al., 2017)	United States of America	Manifestation/incidence/prevalence/ magnitude of ageism	Quantitative - experimental	Cross-sectional	Convenience	125	(M = 27.70, SD = 4.92)	63%	University students	29 years old or a Gen-Y/Millennial vs. 60 vs Baby boomer
(Perry et al., 2017)	United States of America	Manifestation/incidence/prevalence/ magnitude of ageism	Quantitative - experimental	Cross-sectional	Convenience	225	(M= 37.18, SD = 11.36)	54%	Amazon Mechanical Turk (MTurk) users	29 years old or a Gen-Y/Millennial vs. 60 vs Baby boomer
(Perry & Bourhis, 1998)	United States of America	Manifestation/incidence/prevalence/ magnitude of ageism	Quantitative - experimental	Cross-sectional	Convenience	77	19-22	Approx 54.3%	University students	younger job applicants (20,21,24 years old); older job applicants (55,58,60 years old)
(Perry-Hazan, 2016)	Israel	Manifestation/incidence/prevalence/ magnitude of ageism	Qualitative - not specified	Cross-sectional	Purposive or judgement	116 meeting protocols + 4 meetings with 2 boys and 2 girls	17-19 (they were 16-18 when they participated in the meetings)	50%	Young people who participated in policymaking meetings when they were 16-18 years old.	Children and young people
(Petit, 2007)	France	Manifestation/incidence/prevalence/ magnitude of ageism	Quantitative - experimental	Cross-sectional	Convenience	942 applications	Not reported	Not reported	Job offers online	aged 25, single, childless; aged 37, single (or divorced), childless; aged 37, married with three children
(Petrović, Čizmić, & Vukelić, 2018)	Serbia	Manifestation/incidence/prevalence/ magnitude of ageism	Qualitative - content analysis	Cross-sectional	Not reported	50 internship reports	Not reported	Not reported	University students	Young students
(Pietilä, Ojala, King, & Calasanti, 2013)	Finland	Manifestation/incidence/prevalence/ magnitude of ageism	Qualitative - not specified	Cross-sectional	Purposive or judgement	20	24-39	0%	Male industrial workers under 40 years old	All age groups

Author(s), year	Country	Main purpose of the study	Type of Research	Study design timeframe	Sampling approach	Sample size	Age [Range (Mean, SD)]	Sex (% female)	Population	Target age group studied
(Piliavin, 1987)	United States of America	Manifestation/incidence/prevalence/ magnitude of ageism	Quantitative - experimental	Cross-sectional	Convenience	1507	Median=35	52%	Voters	31-year-old white male; 47-year old
(Pinquart Silka Wenzel, 2000)	United States of America	Interventions to tackle ageism	Quantitative - experimental	Cross-sectional	Convenience	20	(M=71.7, SD=8.3 years)	100%	Adults contacted through senior centers	children (8–11 years)
(Posthuma & Campion, 2009)	Not reported	Manifestation/incidence/prevalence/ magnitude of ageism	Literature review	Not relevant	Not clear	117 articles	Not relevant	Not relevant	Relevant articles that met explicit criteria	Young, old
(R. Price, Bailey, McDonald, & Pini, 2011)	Australia	Manifestation/incidence/prevalence/ magnitude of ageism	Qualitative - not specified	Cross-sectional	Purposive or judgement	13	Not reported	Not reported	Individuals who were expected to have an in-depth knowledge of and experience with child labour issues	children
(T. Price & Been, 2018)	United States of America	Interventions to tackle ageism	Qualitative - not specified	Longitudinal	Convenience	9	14 -18	100%	9th-12th grade students	Youth (14-18 years old)
(Priest et al., 2018)	United States of America	Manifestation/incidence/prevalence/ magnitude of ageism	Quantitative - experimental	Cross-sectional	Convenience	1022	18-83 (M=51, SD=15)	64%	Non-Hispanic white civilians who worked and/or volunteered with children.	0-8 years (young children) vs. 13-18 years (teens)
(Rabl & Triana, 2013)	Germany	Consequences of ageism	Quantitative - correlational	Cross-sectional	Convenience	1255 (631 older employees, 624 were younger employees)	30-64 (M=45.11, SD=9.86) Older employees: 50-64 Younger employees: 30-40	44%	Employees	30-40, 50-64
(Range & Goggin, 1990)	United States of America	Manifestation/incidence/prevalence/ magnitude of ageism	Quantitative - experimental	Cross-sectional	Convenience	85	Not reported	Not reported	University students	10,18,30 or 65 years old
(Raymer, Reed, Spiegel, & Purvanova, 2017)	United States of America	Manifestation/incidence/prevalence/ magnitude of ageism	Quantitative - correlational	Cross-sectional	Convenience, Purposive or judgement	282	19-29 years: 19-29: millennials; 30-49: X-ers; 50+: baby boomers	81%	Employees and university students	"The typical young professional"
(Reekie & Hansen, 1992)	United States of America	Determinants of ageism, Manifestation/incidence/prevalence/ magnitude of ageism	Quantitative - experimental	Cross-sectional	Simple Random	103	M=52 women; M=51 men, 26-75	Not reported	Clinical Social Workers	32 vs. 62

Author(s), year	Country	Main purpose of the study	Type of Research	Study design timeframe	Sampling approach	Sample size	Age [Range (Mean, SD)]	Sex (% female)	Population	Target age group studied
(Reno, 1979)	United States of America	Manifestation/incidence/prevalence/ magnitude of ageism	Quantitative - experimental	Cross-sectional	Convenience	93	Females 18-44 (M=25); Males 21-36 (M=26)	74%	University students and teachers	25 vs. 63
(Riach & Rich, 2010)	United Kingdom	Manifestation/incidence/prevalence/ magnitude of ageism	Quantitative - experimental	Cross-sectional	Convenience	234	Not reported	Not reported	Job openings	women aged 21vs 39 years; men aged 27 and 47; women aged 27 vs. 47.
(Riach, 2015)	Germany, France, Spain, United Kingdom	Manifestation/incidence/prevalence/ magnitude of ageism	Quantitative - experimental	Cross-sectional	Not reported	1582 job openings for male waiters in hotels and restaurants across 4 countries (470 in UK; 345 France; 427 Germany; 340 Spain)	Not reported	Not reported	Job openings	aged 27 and 47
(Rogers, Davies, Anderson, & Potton, 2011)	United Kingdom	Determinants of ageism, Manifestation/incidence/prevalence/ magnitude of ageism	Quantitative - experimental	Cross-sectional	Convenience	192	18-90 (M = 38.4; SD = 13.4)	63%	Adults	12 vs 15 years
(Roscoe & Karen, 1989)	United States of America	Manifestation/incidence/prevalence/ magnitude of ageism	Quantitative - descriptive	Cross-sectional	Convenience	95 older adolescents, 78 mothers, 83 grandmothers	Older adolescents<24; Other age groups not reported	100%	3 generations of maternally related females	varying ages were used on items to prevent a response set
(Rosemary & Shobana Nair, 2007)	United Kingdom	Manifestation/incidence/prevalence/ magnitude of ageism	Qualitative - not specified	Cross-sectional	Purposive or judgement	15	Not reported	Not reported	Managers in hospitality firms	Young workers (16-17); Young adult workers (18-21); Older workers (22 and above)
(Ruggs, Hebl, Singletary, Walker, & Fa-Kaji, 2014)	United States of America	Manifestation/incidence/prevalence/ magnitude of ageism	Quantitative - experimental	Cross-sectional	Convenience	320	18-72 (M=35, SD=15)	66%	Not reported	Ages 20 and 40 and 60
(Ryan, King, & Finkelstein, 2015)	United States of America	Consequences of ageism, Manifestation/incidence/prevalence/ magnitude of ageism, Measurement of ageism	Quantitative - correlational	Cross-sectional	Simple Random, Convenience	281	18-30 (M=25.2, SD=3.8)	60%	University students	Younger adults (i.e. members of the participants' age group), older adults
(Salem, Ibrahim, & Brady, 2003)	Egypt	Manifestation/incidence/prevalence/ magnitude of ageism	Qualitative - not specified	Longitudinal	Purposive or judgement	24	18-27 (M-21)	100%	Young rural women who were hired to act as "promoters" in	18-27

Author(s), year	Country	Main purpose of the study	Type of Research	Study design timeframe	Sampling approach	Sample size	Age [Range (Mean, SD)]	Sex (% female)	Population	Target age group studied
									an experimental development program to offer literacy, life skills, and sports activities to disadvantaged out-of-school girls aged 13-15.	
(Santini & Lamura, 2018)	Italy	Interventions to tackle ageism	Qualitative - not specified	Cross-sectional	Convenience	63 (25 14-year old students; 16 older residents; 3 social workers of a residential care facility for older adults; 16 older volunteers; and 3 teachers from a junior secondary school)	students: M=14; volunteers: M=70; older residents: M=83	students: 28%; volunteers: 62.5%; older residents:68.7 %	Secondary school students and older adults from a residential care facility	adolescents; older adults
(Schloegel, Stegmann, van Dick, & Maedche, 2018)	Germany, China, Poland, Bulgaria	Determinants of ageism	Quantitative - correlational	Cross-sectional	Convenience	457	23-63 (M=39, SD=9.98)	Not reported	Employees	younger (<=35 years), middle-aged, and older (>50 years) employees
(Schloegel, Stegmann, Maedche, & van Dick, 2018)	United States of America	Manifestation/incidence/prevalence/ magnitude of ageism	Quantitative - correlational	Cross-sectional	Convenience	464	23-63 (M=38, SD=10.1)	17%	Employees	younger employees (⩽35 years), middle aged employees (36-50), older employees (51+)
(Schniter & Shields, 2014)	United States of America	Manifestation/incidence/prevalence/ magnitude of ageism	Quantitative - experimental	Cross-sectional	Convenience	40	20 younger adults:18-22 (M=18.89, SD=1.1); 20 older adults: 51-84 (M=70.11, SD=10.05)	Younger adults=45%, Older adults=65%	Independently living healthy older adults and university students	Unknown age, the same age group or the other age group
(Schwab & Heneman, 1978)	United States of America	Manifestation/incidence/prevalence/ magnitude of ageism	Quantitative - experimental	Cross-sectional	Convenience	32	(M=34.1, SD = 6.3)	44%	Personnel specialists	24 yrs, 61 yrs
(Selseng, 2015)	Norway	Manifestation/incidence/prevalence/ magnitude of ageism	Qualitative - not specified	Longitudinal	Purposive or judgement	23	20s to 60s	83%	Counsellors working for the Norwegian	Youth

Author(s), year	Country	Main purpose of the study	Type of Research	Study design timeframe	Sampling approach	Sample size	Age [Range (Mean, SD)]	Sex (% female)	Population	Target age group studied
									Labour and Welfare Administration	
(Shabbir et al., 2009)	United Kingdom	Manifestation/incidence/prevalence/ magnitude of ageism	Quantitative - correlational	Longitudinal	Entire population	41	22-49, median 47	61%	Patients <50 diagnosed with colorectal cancer	<50
(Sheahan & Pozzulo, 2017)	United States of America	Manifestation/incidence/prevalence/ magnitude of ageism	Quantitative - experimental	Cross-sectional	Convenience	556	18-46 (M = 20.35, SD = 4.17)	68%	University students	victim age: 12 years vs. 16 years. vs. 20 years; defendant age: 25 years vs. 45 years vs. 65 years
(Shier, Méndez, Centeno, Arróliga, & González, 2014)	Nicaragua	Manifestation/incidence/prevalence/ magnitude of ageism	Qualitative - case study	Cross-sectional	Purposive or judgement	Not clear	Not reported	Not reported	Children and young people who had successfully managed political advocacy	Children and young adults
(Shorel & Bleicken, 1991)	United States of America	Manifestation/incidence/prevalence/ magnitude of ageism	Quantitative - correlational	Cross-sectional	Simple Random	70 (supervisees); 35 (supervisors)	M=35.03 (supervisees); M=36.06 (supervisors)	50% (supervisees); 54.3% (supervisors)	Assemblers	Younger, middle-aged and older subordinates
(Sigelman & Sigelman, 1982)	United States of America	Manifestation/incidence/prevalence/ magnitude of ageism	Quantitative - experimental	Cross-sectional	Convenience	1158	Not reported	42% among Whites; 44% among Blacks	University students	47-year old white or black; 31-year old white or black; 53 year old white ; 72 year old black
(Sikorski, Luppa, Brähler, König, & Riedel-Heller, 2012)	Germany	Manifestation/incidence/prevalence/ magnitude of ageism	Quantitative - experimental	Cross-sectional	Simple Random	3003	18-20 (4.9%), 21-40 (22.4%), 41-60(37.2%), 60-80(31.5%), >81(4%)	52.80%	Civilians	9, 42, 68 year old citizen with obesity
(Silvestre, Huart, & Dardenne, 2017)	France	Manifestation/incidence/prevalence/ magnitude of ageism	Quantitative - experimental	Cross-sectional	Convenience	68	(M=20.90; SD= 3.07)	52.94%	University students	M=20.90
(Silvestre et al., 2017)	France	Manifestation/incidence/prevalence/ magnitude of ageism	Quantitative - experimental	Cross-sectional	Convenience	40	(M=22.05, SD=2.35)	50%	University students	M=22.05
(Silvestre et al., 2017)	France	Manifestation/incidence/prevalence/ magnitude of ageism	Quantitative - experimental	Cross-sectional	Convenience	132	(M=21.53; SD=2.06)	50%	University students	M=21.53
(Singer, 1986)	New Zealand	Determinants of ageism, Manifestation/incidence/prevalence/ magnitude of ageism	Quantitative - correlational	Cross-sectional	Convenience	170	17-19	61.76%	University students	30-year-old, 55-year old

Author(s), year	Country	Main purpose of the study	Type of Research	Study design timeframe	Sampling approach	Sample size	Age [Range (Mean, SD)]	Sex (% female)	Population	Target age group studied
(Singer & Sewell, 1989)	New Zealand	Manifestation/incidence/prevalence/ magnitude of ageism, Determinants of ageism	Quantitative - experimental	Cross-sectional	Convenience	61 managers; 119 students	Managers: M=33; students: M=20	Managers: 27%; students: 59%	University students	"young" (25 years) and "old" (48 years)
(Slevin & Wingrove, 1983)	United States of America	Manifestation/incidence/prevalence/ magnitude of ageism	Quantitative - correlational	Cross-sectional	Convenience	103 (Generation 1; daughters); 88 (Generation 2, mothers); 30 (Generation 3, grandmothers)	18-22 (Generation 1); mid-thirties to upper sixties (Generation 2); 33% of Generation 3 below 60, 43% between 60 and 70, and 24% above 70.	100%	University students	Different family generations
(Snape & Redman, 2003)	United Kingdom	Consequences of ageism, Manifestation/incidence/prevalence/ magnitude of ageism	Quantitative - correlational	Cross-sectional	Stratified	613	18-63 (M=43.22) Under 30 years; 30-39 years; 40-49 years; 50 and older	70%	Employees	Under 30 years; 30-39 years; 40-49 years; 50+
(Song & Zuo, 2016)	China	Manifestation/incidence/prevalence/ magnitude of ageism	Quantitative - experimental	Cross-sectional	Convenience	104	17-23 (M = 19.38, SD = 1.17)	81%	University students	young people vs. older adults
(Song & Zuo, 2016)	China	Manifestation/incidence/prevalence/ magnitude of ageism	Quantitative - experimental	Cross-sectional	Convenience	156	17-27 (M = 19.81, SD = 1.66)	74%	University students	young people vs. older adults
(Souza, 2011)	Brazil	Interventions to tackle ageism	Qualitative - grounded theory	Cross-sectional	Simple Random	32 older adults 111 students	Not reported	Not reported	Students of a secondary school and community dwelling older adults	younger people, older people
(Stewart & Ryan, 1982)	United States of America	Manifestation/incidence/prevalence/ magnitude of ageism	Quantitative - experimental	Cross-sectional	Convenience	60	18-21	68%	University students	younger (20-22 years) or older (60-65 years)
(Stoffers & Van der Heijden, 2018)	Netherlands	Manifestation/incidence/prevalence/ magnitude of ageism	Quantitative - correlational	Cross-sectional	Convenience	487 pairs	Employees: (M=38, SD=11.05); Supervisors: (M=43, SD = 9.23)	Employees: 40%; Supervisors: 18%	Pairs of employees and their immediate supervisors	employees under 40 years, employees over 40, employees over 50
(Sun, Lou Vivian, Dai, To, & Wong Shum, 2019)	China	Interventions to tackle ageism	Quantitative - experimental	Longitudinal	Convenience	73 in the intervention group and 77 older in the comparison group	(M=72.54, SD=7.18) in the intervention group and (M=73.95, SD=8.70) in the comparison group	81% in the intervention group and 79% older in the comparison group	Participants in community social service units for older adults	Young people, older people

Author(s), year	Country	Main purpose of the study	Type of Research	Study design timeframe	Sampling approach	Sample size	Age [Range (Mean, SD)]	Sex (% female)	Population	Target age group studied
(Truxillo & Fraccaroli, 2012)	United States of America	Manifestation/incidence/prevalence/ magnitude of ageism	Quantitative - experimental	Cross-sectional	Convenience	142	19-45 (M=25.7, SD=5.8)	68%	Employed or recently employed university students	24-34 year old; 55-65 year old
(Turner & et al., 1995)	United States of America	Manifestation/incidence/prevalence/ magnitude of ageism	Quantitative - experimental	Cross-sectional	Convenience	671	18-81 (M=31.2, SD=14.9)	74%	University students	late 20s, late 40s
(Uotinen, 1998)	Finland	Manifestation/incidence/prevalence/ magnitude of ageism, Determinants of ageism	Quantitative - correlational	Cross-sectional	Simple Random	446	25-39	58.74%	Community dwelling adults	25-39
(Wagner & Luger, 2017)	United States of America	Measurement of ageism, Manifestation/incidence/prevalence/ magnitude of ageism	Quantitative - correlational	Cross-sectional	Convenience	94 traditional college age adults and 52 older adults	Students: 18-21 (M=18.6); older adults 55-88 (M=70.3)	Students: 77% female; older adults: 58%	University students, older students and older adults in the community	18-25 yrs, 65+
(Walsh & Connor, 1979)	United States of America	Manifestation/incidence/prevalence/ magnitude of ageism	Quantitative - experimental	Cross-sectional	Convenience	74	Not reported	50%	University students	25 vs. 64
(Weinkle & Lee, 2019)	United States of America	Manifestation/incidence/prevalence/ magnitude of ageism	Quantitative - experimental	Cross-sectional	Convenience	184	18-38	77%	University students	younger male (22–24 years old), younger female (24–25 years old), older male or female (in late 60s)
(Weiss & Lang, 2009)	United States of America	Consequences of ageism	Quantitative - experimental	Cross-sectional	Convenience	228	19-82 (M=52.0, SD=17.9)	72%	Adults	young 19-39, middle-aged 41-64, and old adults 65-88
(Williams & Garrett, 2002)	United Kingdom	Manifestation/incidence/prevalence/ magnitude of ageism	Quantitative - correlational	Cross-sectional	Convenience	490	20-29:M=25.97, SD=2.11; 30-39: M=34.10, SD=2.89; 40-49: M=45, SD=2.73; 50-59: M=52.98, sd=2.70	20-29: 51%; 30-39:53%; 40-49: 59%; 50-59: 46%	Community dwelling adults	young teenagers: 13-16; elders: 65-85, participants' own age (groups aged 20-29, 30-39, 40-49)
(Wiseman, 2010)	United States of America	Manifestation/incidence/prevalence/ magnitude of ageism	Quantitative - experimental	Cross-sectional	Convenience	46	Not reported	Not reported	University students	young vs. old
(Wiseman, 2010)	United States of America	Manifestation/incidence/prevalence/ magnitude of ageism	Quantitative - experimental	Cross-sectional	Convenience	40	Not reported	Not reported	University students	young vs. old

Author(s), year	Country	Main purpose of the study	Type of Research	Study design timeframe	Sampling approach	Sample size	Age [Range (Mean, SD)]	Sex (% female)	Population	Target age group studied
(Wiseman, 2007)	United States of America	Manifestation/incidence/prevalence/ magnitude of ageism	Quantitative - experimental	Cross-sectional	Convenience	44	Not reported	Not reported	College students	Young
(Wiseman, 2007)	United States of America	Manifestation/incidence/prevalence/ magnitude of ageism	Quantitative - experimental	Cross-sectional	Convenience	71	Not reported	Not reported	College students	Young
(Worth, 2016)	Canada	Consequences of ageism, Other - individual strategy to confront ageism	Qualitative - not specified	Cross-sectional	Purposive or judgement	33	Not reported	100%	Millennial women	women born in the 1980s
(Zepelin & Heath, 1987)	United States of America	Manifestation/incidence/prevalence/ magnitude of ageism	Quantitative - correlational	Cross-sectional	Quota	462	White color: 18-39: M=26.3 SD=3.4; 40-70: M=55.4 SD=8.7; Blue color:18-39: M=27.8 SD=5.6; 40-70: M=58.7 SD=11.2	50%	White-collar and blue-collar workers	Varied- 21-77
(Zhang & Hummert, 2001)	China	Manifestation/incidence/prevalence/ magnitude of ageism	Qualitative - not specified	Cross-sectional	Convenience	20 younger 13 older	Young M= 24.05, 19-33; Older M=67.10, 62-72	Not reported	College students and community dwelling older adults	younger (18-35) and older adults (55+).
